# The Dependence of Chance-Corrected Weighted Agreement Coefficients on the Power Parameter of the Weighting Scheme: Analysis and Measurement

**DOI:** 10.1007/s11336-022-09881-7

**Published:** 2022-09-06

**Authors:** Rutger van Oest

**Affiliations:** Department of Marketing, BI Norwegian Business School, Nydalsveien 37, 0484 Oslo, Norway

**Keywords:** chance-corrected weighted agreement coefficients, interrater agreement, weighting schemes, power parameter, coefficient susceptibility

## Abstract

We consider the dependence of a broad class of chance-corrected weighted agreement coefficients on the weighting scheme that penalizes rater disagreements. The considered class encompasses many existing coefficients with any number of raters, and one real-valued power parameter defines the weighting scheme that includes linear, quadratic, identity, and radical weights. We obtain the first-order and second-order derivatives of the coefficients with respect to the power parameter and decompose them into components corresponding to all pairs of different category distances. Each component compares its two distances in terms of the ratio of observed to expected-by-chance frequency. A larger ratio for the smaller distance than the larger distance contributes to a positive relationship between the power parameter and the coefficient value; the opposite contributes to a negative relationship. We provide necessary and sufficient conditions for the coefficient value to increase or decrease and the relationship to intensify or weaken as the power parameter increases. We use the first-order and second-order derivatives for corresponding measurement. Furthermore, we show how these two derivatives allow other researchers to obtain quite accurate estimates of the coefficient value for unreported values of the power parameter, even without access to the original data.

**Supplementary Information:**

The online version contains supplementary material available at 10.1007/s11336-022-09881-7.

## Introduction

Agreement coefficients measure the extent to which raters agree when subjectively classifying items into mutually exclusive and exhaustive categories. Examples include the classification of communications based on content, images based on visible aspects, and diagnoses of patients. High rater agreement indicates that the obtained categorical data are reproducible. In contrast, low rater agreement means that the raters interpreted the items or categories differently, jeopardizing the validity of subsequent analyses.

Due to limited choice options, raters may guess the category without knowing the actual category, implying that some rater agreements occur by chance. Because agreements by chance do not provide intrinsic value, agreement coefficients usually aim to exclude them (Banerjee et al. 1999; Janson and Olsson 2001). Different ways to correct for chance agreement have resulted in various agreement coefficients.

In addition to nominal (unordered) categories, many settings involve classification into ordinal (ordered) categories, such as 5-point rating scales. Ordinal categories require the researcher to choose both a suitable agreement coefficient and a weighting scheme that assigns partial credit to rater disagreements. The amount of credit (or penalization) for disagreements typically depends on the distance between the chosen categories, but many options exist to capture this dependence. Before data collection, the researcher should decide on the weighting scheme, and the chosen weights should reflect the specific situation (Cohen 1968; Gwet 2014).

In practice, the two most common weighting schemes are linear and quadratic. The former uses a penalty factor equal to the distance between the chosen categories expressed as a fraction of the maximum possible distance (Cicchetti and Allison 1971); the latter defines the penalty factor by the square of this relative distance (Fleiss and Cohen 1973). The literature has offered coefficient interpretations for linear weights (Kvålseth 2018; Mielke and Berry 2009; Vanbelle and Albert 2009; Warrens 2011), quadratic weights (Cohen 1968; Fleiss and Cohen 1973; Schuster 2004), and both (Vanbelle 2016). Alternatively, radical weights define the penalty factor by the square root of the relative distance (Gwet 2014), and identity weights imply that all possible rater disagreements receive zero credit (i.e., maximum penalization).

Unfortunately, the chosen weighting scheme may substantially affect the coefficient value and thus the conclusion about whether the categorized data are reproducible (Brenner and Kliebsch 1996; Graham and Jackson 1993). For example, it is well known that quadratic weights tend to produce higher coefficient values than linear weights. Indeed, Warrens (2013) computed Cohen’s weighted kappa with linear and quadratic weights for 20 contingency tables from the literature: Quadratic weights resulted in higher coefficient values for 19 out of 20 cases.

Although lenient weighting schemes, such as quadratic weights, are sometimes justified by the specific situation, researchers may abuse such weighting schemes to obtain artificially high scores of interrater agreement. This problem has made some researchers conclude that alternatives with stronger foundations should replace chance-corrected weighted agreement coefficients (e.g., Soeken and Prescott 1986). Although such views may be extreme, they hint at the importance of appropriate measurement and understanding the conditions under which these coefficients are particularly susceptible to the chosen weights and in which direction.

For two-rater coefficients, Warrens (2012a) proved that quadratic weights yield higher coefficient values than linear weights if the contingency table is tridiagonal. Thus, a sufficient condition is that only the main diagonal and the two diagonals immediately next to the main diagonal contain nonzero frequencies, meaning that raters’ chosen categories are at most one step apart. Warrens (2013) provided a generalization of this sufficient condition for two raters: After combining any two diagonals with the same distance to the main diagonal, the ratio of observed to expected-by-chance frequency should decrease as the diagonal’s distance to the main diagonal increases; that is, after scaling by expected frequency, category combinations should occur less often if the categories are farther apart, where combinations with the same distance to the main diagonal compensate for each other. Although this sufficient condition is insightful, violations become increasingly likely as the number of categories (and diagonals) increases.

The present study considers a broad class of chance-corrected weighted agreement coefficients suitable for any number of raters. This class includes weighted versions of existing coefficients, such as the *S*-coefficient, Cohen’s kappa, Scott’s pi, Fleiss’ kappa, and the recently introduced uniform prior coefficient. We relate the coefficient value to one real-valued power parameter that captures common weighting schemes, such as linear, quadratic, identity, and radical weights, and allows for a continuum of infinitely many weighting schemes (Warrens 2014). We derive expressions for the corresponding first-order and second-order derivatives that can act as measures of coefficient susceptibility, which is the direction and degree to which the coefficient value changes as the power parameter of the weighting scheme changes. Next, we show how researchers without access to the original data can use these derivatives to obtain quite accurate estimates of the coefficient value for unreported values of the power parameter (e.g., linear weights when the authors showed only quadratic weights). Finally, we translate the two derivatives into necessary and sufficient conditions for the coefficient value to increase or decrease and the dependence to become stronger or weaker as the power parameter increases. We decompose the derivatives and related conditions into components that pairwise compare all different category distances in terms of the ratio of observed to expected-by-chance frequency.

## Observed Weighted Agreement

Before correcting for chance agreement, we define observed weighted agreement as

1 $$\begin{aligned} A_{w}=\frac{1}{N}\sum \limits _{i=1}^N \left[ \sum \nolimits _{c=1}^C \frac{R_{i,c}(\sum \nolimits _{\tilde{c}=1}^C w_{c,\tilde{c}} R_{i,\tilde{c}}-1)}{R(R-1)} \right] =\frac{\sum \nolimits _{i=1}^N \sum \nolimits _{c=1}^C {R_{i,c}(\sum \limits _{\tilde{c}=1}^C w_{c,\tilde{c}} R_{i,\tilde{c}}-1)} }{NR(R-1)},\, \end{aligned}$$

where *N* is the number of items, $C\ge 3$ is the number of categories, $R\ge 2$ is the number of raters, and $R_{i,c}$ is the number of raters who assign item *i* to category *c*, with $\sum \nolimits _{c=1}^C R_{i,c} =R$. Furthermore, $w_{c,\tilde{c}}$ defines the weights for pairwise rater (dis)agreements, where *c* is the category chosen by the first rater, and $\tilde{c}$ is chosen by the second rater: $w_{c,\tilde{c}}=1$ if $c=\tilde{c}$, and $0\le w_{c,\tilde{c}}<1$ if $c\ne \tilde{c}$ (i.e., full credit if the two raters agree, and partial or no credit if they disagree). Because of symmetric weights, $w_{c,\tilde{c}}=w_{\tilde{c},c}$ for all $c\ne \tilde{c}$. Expression (1) is consistent with Gwet (2014) and Van Oest and Girard (2021); it reduces to Fleiss (1971) if $w_{c,\tilde{c}}=0$ for all $c\ne \tilde{c}$.

As an example, suppose $C=3$ categories, $R=2$ raters, and $N=1$ item for which the first rater chooses category 1 and the second rater chooses category 2. This situation implies that $R_{1,1}=1$, $R_{1,2}=1$, and $R_{1,3}=0$, so $A_{w}=\sum \nolimits _{c=1}^C {R_{1,c}(\sum \nolimits _{\tilde{c}=1}^C w_{c,\tilde{c}} R_{1,\tilde{c}}-1)} /[R(R-1)]$ becomes $\{1\times (w_{1,1}\times 1+w_{1,2}\times 1+w_{1,3}\times 0-1)+1\times (w_{2,1}\times 1+w_{2,2}\times 1+w_{2,3}\times 0-1)+0\times (w_{3,1}\times 1+w_{3,2}\times 1+w_{3,3}\times 0-1)\}/\{2\times (2-1)\}=\{(w_{1,1}+w_{1,2}-1)+(w_{2,1}+w_{2,2}-1)\}/2$. Because $w_{1,1}=1$, $w_{2,2}=1$, and $w_{1,2}=w_{2,1}$ due to symmetry, we obtain $A_{w}=\{(1+w_{1,2}-1)+(w_{1,2}+1-1)\}/2=w_{1,2}$, which is the weight corresponding to the two categories chosen for the item by the rater pair. More generally, $A_{w}$ in (1) is the average value of $w_{c,\tilde{c}}$ across all combinations of items and rater pairs, which reduces to the proportion of pairwise rater agreement if $w_{c,\tilde{c}}=0$ for all $c\ne \tilde{c}$.

We consider weighting schemes that penalize rater disagreements based on the distance between the chosen categories, with power parameter $\gamma$ (Vanbelle 2016; Warrens 2013, 2014):

2 $$\begin{aligned} w_{c,\tilde{c}}=1-\left( \frac{\left| c-\tilde{c} \right| }{C-1} \right) ^{\gamma },\, \gamma >0.\, \end{aligned}$$

These weights become linear if $\gamma =1$, become quadratic if $\gamma =2$, become radical if $\gamma =.5$, and converge to identity weights (i.e., $w_{c,\tilde{c}}=0$ for all $c\ne \tilde{c}$, implying unweighted agreement) if $\gamma \rightarrow 0$. The weighting scheme does not award credit to rater disagreements as $\gamma$ approaches zero, whereas it becomes increasingly lenient as $\gamma$ increases.

A smaller power parameter (e.g., radical weights) is appropriate for situations where even minor rater disagreements are serious. For example, different teachers may need to do grading and independent regrading of student exams, where one-step deviations are part of the game, but larger deviations quickly become unacceptable. Conversely, a larger power parameter (e.g., quadratic weights) is appropriate if only major rater disagreements are problematic. Furthermore, linear weights are suitable if no obvious arguments exist to deviate from penalization in proportion to the distance of disagreement. For the sake of illustration, we write out the weights $W=(w_{c,\tilde{c}})$, defined by (2), for $C=5$ categories and different values of the power parameter:

$$\begin{aligned}&W_{\gamma \rightarrow 0\mathrm {(identity)}}=\left( {\begin{array}{*{20}c} 1 &{} 0 &{} 0 &{} 0 &{} 0\\ 0 &{} 1 &{} 0 &{} 0 &{} 0\\ 0 &{} 0 &{} 1 &{} 0 &{} 0\\ 0 &{} 0 &{} 0 &{} 1 &{} 0\\ 0 &{} 0 &{} 0 &{} 0 &{} 1\\ \end{array} } \right) , \\&W_{\gamma =.5\mathrm {(radical)}}=\left( {\begin{array}{*{20}c} 1 &{} .50 &{} .29 &{} .13 &{} 0\\ .50 &{} 1 &{} .50 &{} .29 &{} .13\\ .29 &{} .50 &{} 1 &{} .50 &{} .29\\ .13 &{} .29 &{} .50 &{} 1 &{} .50\\ 0 &{} .13 &{} .29 &{} .50 &{} 1\\ \end{array} } \right) , \\&W_{\gamma =1\mathrm {(linear)}}=\left( {\begin{array}{*{20}c} 1 &{} .75 &{} .50 &{} .25 &{} 0\\ .75 &{} 1 &{} .75 &{} .50 &{} .25\\ .50 &{} .75 &{} 1 &{} .75 &{} .50\\ .25 &{} .50 &{} .75 &{} 1 &{} .75\\ 0 &{} .25 &{} .50 &{} .75 &{} 1\\ \end{array} } \right) , \\&W_{\gamma =2\mathrm {(quadratic)}}=\left( {\begin{array}{*{20}c} 1 &{} .94 &{} .75 &{} .44 &{} 0\\ .94 &{} 1 &{} .94 &{} .75 &{} .44\\ .75 &{} .94 &{} 1 &{} .94 &{} .75\\ .44 &{} .75 &{} .94 &{} 1 &{} .94\\ 0 &{} .44 &{} .75 &{} .94 &{} 1\\ \end{array} } \right) . \end{aligned}$$

By substituting the weights (2) into observed weighted agreement (1) and subsequently working out the brackets and using that $\left( \left| c-\tilde{c} \right| /(C-1) \right) ^{\gamma }=0$ if $c=\tilde{c}$, we obtain

3 $$\begin{aligned} A_{w}= & {} \frac{\sum \nolimits _{i=1}^N \sum \nolimits _{c=1}^C {\sum \nolimits _{\tilde{c}=1}^C \left[ 1-\left( \frac{\left| c-\tilde{c} \right| }{C-1} \right) ^{\gamma } \right] R_{i,c}R_{i,\tilde{c}}} -\sum \nolimits _{i=1}^N \sum \nolimits _{c=1}^C R_{i,c} }{NR(R-1)}\, \nonumber \\= & {} \frac{NR^{2}-\sum \nolimits _{c=1}^C \sum \nolimits _{\tilde{c}\ne c} {\left( \frac{\left| c-\tilde{c} \right| }{C-1} \right) ^{\gamma }\left[ \sum \nolimits _{i=1}^N {R_{i,c}R_{i,\tilde{c}}} \right] } -NR}{NR(R-1)}\, \nonumber \\= & {} 1-\frac{\sum \nolimits _{c=1}^C \sum \nolimits _{\tilde{c}\ne c} {\left( \frac{\left| c-\tilde{c} \right| }{C-1} \right) ^{\gamma }\left[ \sum \nolimits _{i=1}^N {R_{i,c}R_{i,\tilde{c}}} \right] } }{NR(R-1)}.\, \end{aligned}$$

Symmetry in the numerator of (3) regarding *c* and $\tilde{c}$ (and thus $\tilde{c}<c$ and $\tilde{c}>c$ in $\tilde{c}\ne c)$ implies

4 $$\begin{aligned} A_{w}=1-\sum \limits _{c=2}^C \sum \limits _{\tilde{c}<c} {\left( \frac{c-\tilde{c}}{C-1} \right) ^{\gamma }O_{c,\tilde{c}}} ,\, \end{aligned}$$

where

5 $$\begin{aligned} O_{c,\tilde{c}}=\frac{2\sum \limits _{i=1}^N {R_{i,c}R_{i,\tilde{c}}} }{NR(R-1)}\, \end{aligned}$$

is the *observed* fraction of cases (i.e., combinations of items and rater pairs) for which one rater chooses *c* and the other rater chooses $\tilde{c}<c$. In words, (4) states that observed weighted agreement $A_{w}$ equals one minus the total observed weighted disagreement.

## Chance-Corrected Weighted Agreement

We consider a broad class of chance-corrected weighted agreement coefficients:

6 $$\begin{aligned} I_{w}= & {} \frac{A_{w}-\sum \nolimits _{c=1}^C \sum \nolimits _{\tilde{c}=1}^C {w_{c,\tilde{c}}p_{c}q_{\tilde{c}}} }{1-\sum \nolimits _{c=1}^C \sum \nolimits _{\tilde{c}=1}^C {w_{c,\tilde{c}}p_{c}q_{\tilde{c}}} }\nonumber \\= & {} 1-\frac{1-A_{w}}{1-\sum \nolimits _{c=1}^C \sum \nolimits _{\tilde{c}=1}^C {w_{c,\tilde{c}}p_{c}q_{\tilde{c}}} },\, \end{aligned}$$

where the category proportions in the chance correction sum to one and are greater than zero:

7 $$\begin{aligned} \sum \limits _{c=1}^C p_{c} =\sum \limits _{\tilde{c}=1}^C q_{\tilde{c}} =1,\, p_{c}>0\,\,\, \mathrm { for\,\, }c=1,\ldots ,C,\, q_{\tilde{c}}>0\,\,\, \mathrm {for\,\, }\tilde{c}=1,\ldots ,C.\, \end{aligned}$$

The first part of (7) is logical consistency; the two other parts hold if, for example, all categories are chosen at least once by one of the raters if $p_{c}=q_{c}$ for all *c* or chosen by any two raters (not necessarily for the same item) if $p_{c}\ne q_{c}$. The class of coefficients, defined by (6) and (7), is general. For $R=2$ raters, it includes weighted versions of Cohen’s kappa (Cohen 1960, 1968) and Scott’s pi (Scott 1955). For $R\ge 2$ raters, it includes weighted versions of Fleiss’ kappa (Fleiss 1971), the uniform prior coefficient (Van Oest 2019; Van Oest and Girard 2021), and the *S*-coefficient (Bennett et al. 1954; Brennan and Prediger 1981; Warrens 2014). Table 1 provides the operationalizations of $p_{c}$ and $q_{c}$ for these coefficients.

Although the class of coefficients does not include Krippendorff’s alpha (Gwet, 2014, p.88), this coefficient converges to the weighted Fleiss’ kappa as the number of items *N* increases. Thus, these coefficients usually provide similar values (Gwet 2014). Another excluded coefficient is the weighted kappa for $R\ge 3$ raters (Mielke et al. 2007, 2009). This coefficient considers the *R*-dimensional category combinations from all raters together (instead of rater pairs) but is equivalent to a weighted version of Conger’s kappa, where expected weighted agreement $\sum \nolimits _{c=1}^C \sum \nolimits _{\tilde{c}=1}^C {w_{c,\tilde{c}}p_{c}q_{\tilde{c}}}$ becomes the corresponding average across all rater pairs, with rater-specific category proportions $p_{c}$ and $q_{\tilde{c}}$ (Conger 1980; Warrens 2012b). Furthermore, the class of coefficients excludes Gwet’s AC2 (Gwet, 2014, p.89), which replaces $\sum \nolimits _{c=1}^C \sum \nolimits _{\tilde{c}=1}^C {w_{c,\tilde{c}}p_{c}q_{\tilde{c}}}$ by a substantially different expression.

We substitute the weights (2) and observed weighted agreement (4) into coefficient (6):

8 $$\begin{aligned} I_{w}= & {} 1-\frac{\sum \nolimits _{c=2}^C \sum \nolimits _{\tilde{c}<c} {\left( \frac{c-\tilde{c}}{C-1} \right) ^{\gamma }O_{c,\tilde{c}}} }{1-\sum \nolimits _{c=1}^C \sum \nolimits _{\tilde{c}=1}^C {\left[ 1-\left( \frac{\left| c-\tilde{c} \right| }{C-1} \right) ^{\gamma } \right] p_{c}q_{\tilde{c}}} }\,\nonumber \\= & {} 1-\frac{\sum \nolimits _{c=2}^C \sum \nolimits _{\tilde{c}<c} {\left( \frac{c-\tilde{c}}{C-1} \right) ^{\gamma }O_{c,\tilde{c}}} }{1-\sum \nolimits _{c=1}^C \sum \nolimits _{\tilde{c}=1}^C {p_{c}q_{\tilde{c}}} +\sum \nolimits _{c=1}^C \sum \nolimits _{\tilde{c}=1}^C {\left( \frac{\left| c-\tilde{c} \right| }{C-1} \right) ^{\gamma }p_{c}q_{\tilde{c}}} }\nonumber \\= & {} 1-\frac{\sum \nolimits _{c=2}^C \sum \nolimits _{\tilde{c}<c} {\left( c-\tilde{c} \right) ^{\gamma }O_{c,\tilde{c}}} }{\sum \nolimits _{c=2}^C \sum \nolimits _{\tilde{c}<c} {\left( c-\tilde{c} \right) ^{\gamma }E_{c,\tilde{c}}} },\, \end{aligned}$$

where $\sum \nolimits _{c=1}^C \sum \nolimits _{\tilde{c}=1}^C {p_{c}q_{\tilde{c}}} =1$ in the middle step of (8) due to property (7), and

9 $$\begin{aligned} E_{c,\tilde{c}}=p_{c}q_{\tilde{c}}+q_{c}p_{\tilde{c}}\, \end{aligned}$$

is the fraction of cases (i.e., combinations of items and rater pairs) *expected by chance* for which one rater chooses *c* and the other rater chooses $\tilde{c}<c$, with $E_{c,\tilde{c}}>0$ because of (7). Coefficient $I_{w}$ in (8) equals one minus the ratio of the total observed and expected weighted disagreements.

To facilitate further interpretation, we recall the definition of $O_{c,\tilde{c}}$ in (5) and define

10 $$\begin{aligned} O_{(l)}=\sum \limits _{c=2}^C \sum \limits _{\tilde{c}<c:c-\tilde{c}=l} O_{c,\tilde{c}} \, \end{aligned}$$

as the *observed* fraction of cases for which the categories *c* and $\tilde{c}<c$, chosen by two raters, are $l\in \{1,\ldots ,C-1\}$ steps apart; we put brackets around subscript *l* to emphasize that it refers to the distance between categories. Analogously, recalling the definition of $E_{c,\tilde{c}}$ in (9), we define

11 $$\begin{aligned} E_{(l)}=\sum \limits _{c=2}^C \sum \limits _{\tilde{c}<c:c-\tilde{c}=l} E_{c,\tilde{c}} \, \end{aligned}$$

as the fraction of cases *expected by chance* for which the categories *c* and $\tilde{c}<c$, obtained from two raters, are *l* steps apart. Table 1 provides the exact expressions of (11) for the coefficients. We note that $E_{(l)}>0$ because of (7). Using (10) and (11), we rewrite the chance-corrected weighted agreement coefficient (8) in terms of all possible category distances and their observed and expected frequencies:

12 $$\begin{aligned} I_{w}=1-\frac{\sum \nolimits _{l=1}^{C-1} {l^{\gamma }O_{\left( l \right) }} }{\sum \nolimits _{l=1}^{C-1} {l^{\gamma }E_{\left( l \right) }} }.\, \end{aligned}$$

**Table 1:** Operationalizations of coefficients within considered class.

Coefficient	*R*	$p_{c}$	$q_{c}$	$E_{(l)}=\sum \limits _{c=2}^C \sum \limits _{\tilde{c}<c:c-\tilde{c}=l} {(p_{c}q_{\tilde{c}}+q_{c}p_{\tilde{c}})}$
$S-$coefficient	$\ge 2$	$\frac{1}{C}$	$\frac{1}{C}$	$\frac{2(C-l)}{C^{2}}$
Cohen’s kappa	2	$\frac{\sum \limits _{i=1}^N R_{i,c}^{(1)} }{N}$	$\frac{\sum \limits _{i=1}^N R_{i,c}^{(2)} }{N}$	$\sum \limits _{c=2}^C \sum \limits _{\tilde{c}<c:c-\tilde{c}=l} \left[ \left( \frac{\sum \limits _{i=1}^N R_{i,c}^{(1)} }{N} \right) \left( \frac{\sum \limits _{i=1}^N R_{i,\tilde{c}}^{(2)} }{N} \right) +\left( \frac{\sum \limits _{i=1}^N R_{i,c}^{(2)} }{N} \right) \left( \frac{\sum \limits _{i=1}^N R_{i,\tilde{c}}^{(1)} }{N} \right) \right]$
Scott’s pi	2	$\frac{\sum \limits _{i=1}^N {(R_{i,c}^{\left( 1 \right) }+R_{i,c}^{\left( 2 \right) })} }{2N}$	$\frac{\sum \limits _{i=1}^N {(R_{i,c}^{\left( 1 \right) }+R_{i,c}^{\left( 2 \right) })} }{2N}$	$\sum \limits _{c=2}^C \sum \limits _{\tilde{c}<c:c-\tilde{c}=l} \left[ 2\left( \frac{\sum \limits _{i=1}^N {(R_{i,c}^{\left( 1 \right) }+R_{i,c}^{\left( 2 \right) })} }{2N} \right) \left( \frac{\sum \limits _{i=1}^N {(R_{i,\tilde{c}}^{\left( 1 \right) }+R_{i,\tilde{c}}^{\left( 2 \right) })} }{2N} \right) \right]$
Fleiss’ kappa	$\ge 2$	$\frac{\sum \limits _{i=1}^N R_{i,c} }{NR}$	$\frac{\sum \limits _{i=1}^N R_{i,c} }{NR}$	$\sum \limits _{c=2}^C \sum \limits _{\tilde{c}<c:c-\tilde{c}=l} \left[ 2\left( \frac{\sum \limits _{i=1}^N R_{i,c} }{NR} \right) \left( \frac{\sum \limits _{i=1}^N R_{i,\tilde{c}} }{NR} \right) \right]$
Uniform prior	$\ge 2$	$\frac{1+\sum \limits _{i=1}^N R_{i,c} }{C+NR}$	$\frac{1+\sum \limits _{i=1}^N R_{i,c} }{C+NR}$	$\sum \limits _{c=2}^C \sum \limits _{\tilde{c}<c:c-\tilde{c}=l} \left[ 2\left( \frac{1+\sum \limits _{i=1}^N R_{i,c} }{C+NR} \right) \left( \frac{1+\sum \limits _{i=1}^N R_{i,\tilde{c}} }{C+NR} \right) \right]$

*C* is the number of categories, $C-l$ is the number of combinations $(c,\tilde{c})$ with $\tilde{c}<c$ and $c-\tilde{c}=l$, *N* is the number of items, *R* is the number of raters, $R_{i,c}$ is the number of raters who assign item *i* to category *c*, $R_{i,c}^{(1)}=1$ if rater 1 assigns item *i* to category *c* (zero else), and $R_{i,c}^{(2)}=1$ if rater 2 assigns item *i* to category *c* (zero else). For later reference: Theorems 1 and 2, and Corollaries 1, 2, 3, 7, and 8 apply to all coefficients, with Corollaries 2 and 8 pertaining to $C=3$ categories. Furthermore, Corollaries 4, 5, 6, and 9 specifically apply to the *S*-coefficient, with Corollaries 5 and 9 pertaining to $C=3$ categories.

## First-Order Derivative

The first-order derivative of $I_{w}$ with respect to $\gamma$ describes coefficient susceptibility, that is, the direction and degree to which the coefficient value changes as the power parameter of the weighting scheme increases.

### Theorem 1

The first-order derivative of coefficient $I_{w}$ in (12) with respect to power parameter $\gamma$ in weighting scheme (2) is

13 $$\begin{aligned} \frac{dI_{w}}{d\gamma }=\sum \limits _{m=2}^{C-1} \sum \limits _{l<m} {\mathrm {ln}\left( \frac{m}{l} \right) \left( \frac{l^{\gamma }E_{\left( l \right) }}{\sum \limits _{s=1}^{C-1} {s^{\gamma }E_{\left( s \right) }} } \right) \left( \frac{m^{\gamma }E_{\left( m \right) }}{\sum \limits _{s=1}^{C-1} {s^{\gamma }E_{\left( s \right) }} } \right) \left\{ \frac{O_{\left( l \right) }}{E_{\left( l \right) }}-\frac{O_{\left( m \right) }}{E_{\left( m \right) }} \right\} } ,\, \end{aligned}$$

where we obtained coefficient (12) from (1), (2), (6), and (7), the summation indices l, m, and s are category distances, $O_{\left( l \right) }$ is defined by (10) and (5), and $E_{\left( l \right) }$ is defined by (11) and (9).

### Proof

Differentiating (12) with respect to $\gamma$ yields

$$\begin{aligned} \, \frac{dI_{w}}{d\gamma }= & {} \frac{d\left( 1-\frac{\sum \nolimits _{l=1}^{C-1} {l^{\gamma }O_{\left( l \right) }} }{\sum \nolimits _{l=1}^{C-1} {l^{\gamma }E_{\left( l \right) }} } \right) }{d\gamma } \\= & {} \frac{-\, \left\{ \sum \nolimits _{l=1}^{C-1} {l^{\gamma }\ln \left( l \right) O_{\left( l \right) }} \right\} \left\{ \sum \nolimits _{m=1}^{C-1} {m^{\gamma }E_{\left( m \right) }} \right\} +\left\{ \sum \nolimits _{l=1}^{C-1} {l^{\gamma }O_{\left( l \right) }} \right\} \left\{ \sum \nolimits _{m=1}^{C-1} {m^{\gamma }\ln \left( m \right) E_{\left( m \right) }} \right\} }{\left( \sum \nolimits _{s=1}^{C-1} {s^{\gamma }E_{\left( s \right) }} \right) ^{2}} \\= & {} \frac{-\sum \nolimits _{m=1}^{C-1} \sum \nolimits _{l=1}^{C-1} {\ln \left( l \right) l^{\gamma }m^{\gamma }O_{\left( l \right) }E_{\left( m \right) }} +\sum \nolimits _{m=1}^{C-1} \sum \nolimits _{l=1}^{C-1} {\ln \left( m \right) l^{\gamma }m^{\gamma }O_{\left( l \right) }E_{\left( m \right) }} }{\left( \sum \nolimits _{s=1}^{C-1} {s^{\gamma }E_{\left( s \right) }} \right) ^{2}},\, \end{aligned}$$

where the notation uses different indices *l*, *m*, and *s* in the summations to allow for combining. Using that $-\ln \left( l \right) +\mathrm {ln}\left( m \right) =\mathrm {ln}(m/l)$ and that $\mathrm {ln}(m/l)=0$ if $l=m$, we obtain

$$\begin{aligned} \frac{dI_{w}}{d\gamma }=\left( \sum \limits _{m=1}^{C-1} \sum \limits _{l=1,l\ne m}^{C-1} {\mathrm {ln}\left( \frac{m}{l} \right) l^{\gamma }m^{\gamma }O_{\left( l \right) }E_{\left( m \right) }} \right) /\left( \sum \limits _{s=1}^{C-1} {s^{\gamma }E_{\left( s \right) }} \right) ^{2}.\, \end{aligned}$$

Next, we decompose $l\ne m$ into $l<m$ and $l>m$:

$$\begin{aligned} \frac{dI_{w}}{d\gamma }= & {} \left( \sum \limits _{m=1}^{C-1} \sum \limits _{l<m} {\mathrm {ln}\left( \frac{m}{l} \right) l^{\gamma }m^{\gamma }O_{\left( l \right) }E_{\left( m \right) }} +\sum \limits _{m=1}^{C-1} \sum \limits _{l>m} {\mathrm {ln}\left( \frac{m}{l} \right) l^{\gamma }m^{\gamma }O_{\left( l \right) }E_{\left( m \right) }} \right) /\left( \sum \limits _{s=1}^{C-1} {s^{\gamma }E_{\left( s \right) }} \right) ^{2}\\= & {} \left( \sum \limits _{m=1}^{C-1} \sum \limits _{l<m} {\mathrm {ln}\left( \frac{m}{l} \right) l^{\gamma }m^{\gamma }O_{\left( l \right) }E_{\left( m \right) }} +\sum \limits _{m=1}^{C-1} \sum \limits _{l<m} {\mathrm {ln}\left( \frac{l}{m} \right) m^{\gamma }l^{\gamma }O_{\left( m \right) }E_{\left( l \right) }} \right) /\left( \sum \limits _{s=1}^{C-1} {s^{\gamma }E_{\left( s \right) }} \right) ^{2} \\= & {} \left( \sum \limits _{m=1}^{C-1} \sum \limits _{l<m} {\mathrm {ln}\left( \frac{m}{l} \right) l^{\gamma }m^{\gamma }\left\{ O_{\left( l \right) }E_{\left( m \right) }-O_{\left( m \right) }E_{\left( l \right) } \right\} } \right) /\left( \sum \limits _{s=1}^{C-1} {s^{\gamma }E_{\left( s \right) }} \right) ^{2}.\, \end{aligned}$$

Using that $m=1$ is infeasible if $l<m$, and rewriting yields the result, completing the proof. $\square$

The first-order derivative in (13) is a weighted sum taken over all pairs of different category distances *m* and $l<m$. As reflected by the term $(O_{\left( l \right) }/E_{\left( l \right) })-\mathrm {\, }(O_{\left( m \right) }/E_{\left( m \right) })$, each component compares its smaller distance *l* with its larger distance *m* in terms of the ratio of observed to expected-by-chance frequency. Because all other terms in (13) are strictly positive, it holds that $dI_{w}/d\gamma >0$ (i.e., coefficient $I_{w}$ is increasing in power parameter $\gamma )$ if the ratio of observed to expected-by-chance frequency tends to decrease as categories are farther apart; that is, if mostly $O_{\left( l \right) }/E_{\left( l \right) }>O_{\left( m \right) }/E_{\left( m \right) }$ for $l<m$. However, violations are allowed because of the compensatory structure in the weighted sum.

In (13), a component’s comparison of category distances becomes more important in shaping the first-order derivative as (i) the ratio of the larger versus smaller distance increases (via $\mathrm {ln}\left( m/l \right) )$, and (ii) these two distances capture greater shares of the total expected weighted disagreement across all distances (via the fractions $l^{\gamma }E_{\left( l \right) }/(\sum \limits _{s=1}^{C-1} {s^{\gamma }E_{\left( s \right) }} )$ and $m^{\gamma }E_{\left( m \right) }/(\sum \limits _{s=1}^{C-1} {s^{\gamma }E_{\left( s \right) }} ))$. Furthermore, the latter implies that a component’s importance increases as its two distances *l* and *m* are more likely to occur by chance (via $E_{\left( l \right) }$ and $E_{\left( m \right) })$, and these distances *l* and *m* increase, where higher values of $\gamma$ play a reinforcing role (via $l^{\gamma }$ and $m^{\gamma })$. For example, in a two-rater contingency table, the elements far from the main diagonal increasingly determine how the coefficient value responds to changes in the power parameter as this parameter increases. An implication is that the relationship between the power parameter and the coefficient value can be non-monotonic, as changes in $\gamma$ trigger shifts in the importance of components that compare different category distances, with possibly opposite contributions via the signs of $(O_{\left( l \right) }/E_{\left( l \right) })-\mathrm {\, }(O_{\left( m \right) }/E_{\left( m \right) })$. Furthermore, the log-ratio of category distances $\mathrm {ln}(m/l)$ in (13) implies that the degree of coefficient susceptibility is often higher in settings with more categories (i.e., higher *C*). The reason is that $\mathrm {ln}(m/l)$ tends to take higher values as *C* increases, magnifying the effects of the comparisons $(O_{\left( l \right) }/E_{\left( l \right) })-\mathrm {\, }(O_{\left( m \right) }/E_{\left( m \right) })$.

## Conditions for Direction of Coefficient Susceptibility

We obtain a necessary and sufficient condition from (13) in Theorem 1:

### Corollary 1a

*As power parameter* $\gamma$ in weighting scheme (2) *increases, the chance-corrected weighted agreement coefficient* $I_{w}$ in (12) *increases if and only if*

$$\begin{aligned} \sum \limits _{m=2}^{C-1} \sum \limits _{l<m} {\mathrm {ln}\left( \frac{m}{l} \right) l^{\gamma }m^{\gamma }E_{\left( l \right) }E_{\left( m \right) }\left\{ \frac{O_{\left( l \right) }}{E_{\left( l \right) }}-\frac{O_{\left( m \right) }}{E_{\left( m \right) }} \right\} } >0. \end{aligned}$$

### Corollary 1b

*As power parameter* $\gamma$ *in weighting scheme* (2) *increases, the chance-corrected weighted agreement coefficient* $I_{w}$ in (12) *decreases if and only if*

$$\begin{aligned} \sum \limits _{m=2}^{C-1} \sum \limits _{l<m} {\mathrm {ln}\left( \frac{m}{l} \right) l^{\gamma }m^{\gamma }E_{\left( l \right) }E_{\left( m \right) }\left\{ \frac{O_{\left( l \right) }}{E_{\left( l \right) }}-\frac{O_{\left( m \right) }}{E_{\left( m \right) }} \right\} } <0. \end{aligned}$$

### Proof

We have $\sum \nolimits _{s=1}^{C-1} {s^{\gamma }E_{\left( s \right) }} >0$ due to property (7), so we may ignore these $\sum \nolimits _{s=1}^{C-1} {s^{\gamma }E_{\left( s \right) }}$ terms that do not determine the sign of $dI_{w}/d\gamma$ in (13). Thus, $dI_{w}/d\gamma >0$ is equivalent to the simpler condition in Corollary 1a, and $dI_{w}/d\gamma <0$ is equivalent to the condition in Corollary 1b. $\square$

As before, each component compares its two category distances *m* and $l<m$ in terms of the ratio of observed to expected-by-chance frequency, and components comparing larger distances become relatively more important as the power parameter increases (via $l^{\gamma }$ and $m^{\gamma })$.

For $C=3$ categories, the necessary and sufficient condition in Corollary 1 becomes particularly simple:

### Corollary 2a

As power parameter $\gamma$ in weighting scheme (2) increases in settings with three categories, the chance-corrected weighted agreement coefficient $I_{w}$ in (12) increases if and only if

$$\begin{aligned} O_{\left( 1 \right) }/E_{\left( 1 \right) }>O_{\left( 2 \right) }/E_{\left( 2 \right) }{.} \end{aligned}$$

### Corollary 2b

As power parameter $\gamma$ *in weighting scheme* (2) *increases in settings with three categories, the chance-corrected weighted agreement coefficient* $I_{w}$ *in* (12) *decreases if and only if*

$$\begin{aligned} O_{\left( 1 \right) }/E_{\left( 1 \right) }<O_{\left( 2 \right) }/E_{\left( 2 \right) }{.} \end{aligned}$$

### Proof

The only feasible pair of category distances with $l<m$ for $C=3$ categories corresponds to $l=1$ and $m=2$. Because $\mathrm {ln}\left( m/l \right) l^{\gamma }m^{\gamma }E_{\left( l \right) }E_{\left( m \right) }>0$, substituting $l=1$ and $m=2$ into Corollary 1 yields Corollary 2.

Corollary 2 implies that the relationship between $\gamma$ and $I_{w}$ is always monotonic (either increasing or decreasing) for $C=3$ categories. The direction is determined by whether the ratio of observed to expected-by-chance frequency is greater for combinations of categories that are one step apart or two steps apart.

Furthermore, we obtain a sufficient condition that extends the sufficient condition by Warrens (2013) beyond two raters:

### Corollary 3a

As power parameter $\gamma$ in weighting scheme (2) increases, the chance-corrected weighted agreement coefficient $I_{w}$ in (12) increases if the ratio $O_{(l)}/E_{(l)}$ is decreasing in the category distance l.

### Corollary 3b

As power parameter $\gamma$ in weighting scheme (2) *increases, the chance-corrected weighted agreement coefficient*$I_{w}$ in (12) *decreases if the ratio* $O_{(l)}/E_{(l)}$ *is increasing in the category distance l*.

### Proof

Because $\mathrm {ln}\left( m/l \right) l^{\gamma }m^{\gamma }E_{\left( l \right) }E_{\left( m \right) }>0$, the necessary and sufficient condition for $dI_{w}/d\gamma >0$ in Corollary 1a is satisfied if $(O_{(l)}/E_{(l)})-(O_{(m)}/E_{(m)})>0$ for all $l<m$; that is, if $O_{(l)}/E_{(l)}$ is decreasing in *l*. Similarly, the necessary and sufficient condition for $dI_{w}/d\gamma <0$ in Corollary 1b is satisfied if $(O_{(l)}/E_{(l)})-(O_{(m)}/E_{(m)})<0$ for all $l<m$; that is, if $O_{(l)}/E_{(l)}$ is increasing in *l*. $\square$

Thus, the relationship between power parameter $\gamma$ and coefficient $I_{w}$ is monotonic if the ratio of observed to expected-by-chance frequency is monotonic in the category distance. The sufficient condition in Corollary 3 is both necessary and sufficient in three-category settings due to Corollary 2.

## Conditions for Direction of Coefficient Susceptibility: Weighted *S*-coefficient

It is instructive to apply the three corollaries to the weighted *S*-coefficient, which assumes that all *C* categories are equally likely to occur by chance; that is, $p_{c}=q_{c}=1/C$, $c=1,\ldots ,C$, and hence $E_{\left( l \right) }=2(C-l)/C^{2}$ (see Table 1). We note that $C-l$ is the number of category combinations $(c,\tilde{c})$ with distance *l*; that is, satisfying $c-\tilde{c}=l$. By applying Corollary 1 to the weighted *S*-coefficient, we obtain a necessary and sufficient condition for this coefficient:

### Corollary 4a

As power parameter $\gamma$ *in weighting scheme* (2) *increases, the weighted S-coefficient increases if and only if*

$$\begin{aligned} \sum \limits _{m=2}^{C-1} \sum \limits _{l<m} {\mathrm {ln}\left( \frac{m}{l} \right) l^{\gamma }m^{\gamma }(C-l)(C-m)\left\{ \, \frac{O_{\left( l \right) }}{C-l}-\frac{O_{\left( m \right) }}{C-m}\, \right\} } >0. \end{aligned}$$

### Corollary 4b

As power parameter $\gamma$ *in weighting scheme* (2) *increases, the weighted S-coefficient decreases if and only if*

$$\begin{aligned} \sum \limits _{m=2}^{C-1} \sum \limits _{l<m} {\mathrm {ln}\left( \frac{m}{l} \right) l^{\gamma }m^{\gamma }(C-l)(C-m)\left\{ \, \frac{O_{\left( l \right) }}{C-l}-\frac{O_{\left( m \right) }}{C-m}\, \right\} } <0. \end{aligned}$$

### Proof

Substituting $E_{\left( l \right) }=2(C-l)/C^{2}$ into Corollary 1 and ignoring the positive constant term 2/$C^{2}$ (that does not affect the sign) yields the result. $\square$

As before, this condition considers a weighted sum taken over all pairs of different category distances *m* and $$l<m$$. As reflected by the term $$(O_{\left( l \right) }/(C-l))-(O_{\left( m \right) }/(C-m))$$, each component compares its smaller distance *l* with its larger distance *m* in terms of the average observed frequency per category combination $$(c,\tilde{c})$$. Component importance increases as more category combinations $$(c,\tilde{c})$$ have the corresponding distances *l* and *m* (via $$C-l$$ and $$C-m)$$, the ratio of the larger versus smaller distance increases (via $$\mathrm {ln}\left( m/l \right) )$$, and these distances themselves increase, where higher values of $$\gamma $$ play a reinforcing role (via $$l^{\gamma }$$ and $$m^{\gamma })$$.

Analogous to Corollary 2, the necessary and sufficient condition for the weighted *S*-coefficient in Corollary 4 becomes particularly simple if there are only $C=3$ categories:

### Corollary 5a

As power parameter $\gamma$ *in weighting scheme* (2) *increases in settings with three categories, the weighted S-coefficient increases if and only if* $O_{\left( 1 \right) }/2>O_{\left( 2 \right) }$.

### Corollary 5b

As power parameter $\gamma$ in weighting scheme (2) increases in settings with three categories, the weighted S-coefficient decreases if and only if $O_{\left( 1 \right) }/2<O_{\left( 2 \right) }$.

### Proof

The only feasible pair of category distances with $l<m$ for $C=3$ categories corresponds to $l=1$ and $m=2$. Because $\mathrm {ln}\left( m/l \right) l^{\gamma }m^{\gamma }\left( C-l \right) \left( C-m \right) >0$, substituting $l=1$, $m=2$, and $C=3$ into Corollary 4 yields Corollary 5. $\square$

Corollary 5 implies that the relationship between the power parameter and the weighted *S*-

coefficient is always monotonic (either increasing or decreasing) for $C=3$ categories. The direction is determined by whether the average observed frequency of the two category combinations $(c=2,\tilde{c}=1)$ and $(c=3,\tilde{c}=2)$, with distance one, is greater than the observed frequency of category combination $(c=3,\tilde{c}=1)$, with distance two, or not.

Furthermore, we obtain a sufficient condition for the weighted *S*-coefficient that extends a sufficient condition by Warrens (2014) beyond two raters:

### Corollary 6a

As power parameter $\gamma$ *in weighting scheme* (2) *increases, the weighted S-coefficient increases if the average observed frequency* $O_{(l)}/(C-l)$ *is decreasing in distance l*.

### Corollary 6b

As power parameter $\gamma$ *in weighting scheme* (2) *increases, the weighted S-coefficient decreases if the average observed frequency* $O_{(l)}/(C-l)$ is increasing in distance l.

### Proof

Because $\mathrm {ln}(m/l)l^{\gamma }m^{\gamma }(C-l)(C-m)>0$, the necessary and sufficient condition for $dI_{w}/d\gamma >0$ in Corollary 4a is satisfied if $(O_{(l)}/(C-l))-(O_{(m)}/(C-m))>0$ for all $l<m$; that is, if $O_{(l)}/(C-l)$ is decreasing in *l*. Similarly, the necessary and sufficient condition for $dI_{w}/d\gamma <0$ in Corollary 4b is satisfied if $(O_{(l)}/(C-l))-(O_{(m)}/(C-m))<0$ for all $l<m$; that is, if $O_{(l)}/(C-l)$ is increasing in *l*. $\square$

Thus, the relationship between the power parameter and the weighted *S*-coefficient is monotonic if the average observed frequency per category combination is monotonic in the category distance. The sufficient condition in Corollary 6 is both necessary and sufficient in three-category settings due to Corollary 5.

## Second-Order Derivative

The second-order derivative of $I_{w}$ with respect to $\gamma$ helps describe change in coefficient susceptibility, that is, whether the coefficient’s susceptibility to the power parameter of the weighting scheme intensifies or weakens as this parameter increases.

### Theorem 2

The second-order derivative of coefficient $I_{w}$ in (12) with respect to power parameter $\gamma$ in weighting scheme (2) is

14 $$\begin{aligned} \frac{d^{2}I_{w}}{{(d\gamma )}^{2}}=\sum \limits _{m=2}^{C-1} \sum \limits _{l<m} {\mathrm {ln}\left( \frac{m}{l} \right) \left( \frac{l^{\gamma }E_{\left( l \right) }}{\sum \limits _{s=1}^{C-1} {s^{\gamma }E_{\left( s \right) }} } \right) \left( \frac{m^{\gamma }E_{\left( m \right) }}{\sum \limits _{s=1}^{C-1} {s^{\gamma }E_{\left( s \right) }} } \right) \left\{ \frac{\sum \limits _{s=1}^{C-1} {\mathrm {ln(}lm\mathrm {/}s^{2}\mathrm {)}s^{\gamma }E_{\left( s \right) }} }{\sum \limits _{s=1}^{C-1} {s^{\gamma }E_{\left( s \right) }} } \right\} \left\{ \frac{O_{\left( l \right) }}{E_{\left( l \right) }}-\frac{O_{\left( m \right) }}{E_{\left( m \right) }} \right\} } .\nonumber \\ \end{aligned}$$

### Proof

Starting from the first-order derivative in (13), we first note that

$$\begin{aligned} \frac{d\left( \frac{l^{\gamma }E_{\left( l \right) }}{\sum \limits _{s=1}^{C-1} {s^{\gamma }E_{\left( s \right) }} } \right) }{d\gamma }= & {} \frac{l^{\gamma }\mathrm {ln} \left( l \right) E_{\left( l \right) }\sum \limits _{s=1}^{C-1} {s^{\gamma }E_{\left( s \right) }} -l^{\gamma }E_{\left( l \right) }\sum \limits _{s=1}^{C-1} {s^{\gamma }\ln \left( s \right) E_{\left( s \right) }} }{\left( \sum \limits _{s=1}^{C-1} {s^{\gamma }E_{\left( s \right) }} \right) ^{2}} \\= & {} \left( \frac{l^{\gamma }E_{\left( l \right) }}{\sum \limits _{s=1}^{C-1} {s^{\gamma }E_{\left( s \right) }} } \right) \left\{ \mathrm {ln}\left( l \right) -\frac{\sum \limits _{s=1}^{C-1} {\mathrm {ln}(s)s^{\gamma }E_{\left( s \right) }} }{\sum \limits _{s=1}^{C-1} {s^{\gamma }E_{\left( s \right) }} } \right\} , \end{aligned}$$

so that

$$\begin{aligned}&\frac{d\left( \frac{l^{\gamma }E_{\left( l \right) }}{\sum \limits _{s=1}^{C-1} {s^{\gamma }E_{\left( s \right) }} } \right) \left( \frac{m^{\gamma }E_{\left( m \right) }}{\sum \limits _{s=1}^{C-1} {s^{\gamma }E_{\left( s \right) }} } \right) }{d\gamma }\\&\quad =\left( \frac{l^{\gamma }E_{\left( l \right) }}{\sum \limits _{s=1}^{C-1} {s^{\gamma }E_{\left( s \right) }} } \right) \left( \frac{m^{\gamma }E_{\left( m \right) }}{\sum \limits _{s=1}^{C-1} {s^{\gamma }E_{\left( s \right) }} } \right) \left\{ \mathrm {ln}\left( lm \right) -2\frac{\sum \limits _{s=1}^{C-1} {\mathrm {ln}(s)s^{\gamma }E_{\left( s \right) }} }{\sum \limits _{s=1}^{C-1} {s^{\gamma }E_{\left( s \right) }} } \right\} . \end{aligned}$$

Using this result and (13), we obtain

$$\begin{aligned} \frac{d^{2}I_{w}}{\left( d\gamma \right) ^{2}}&=\frac{d\left( \frac{dI_{w}}{d\gamma } \right) }{d\gamma } =\sum \limits _{m=2}^{C-1} \sum \limits _{l<m} {\, \mathrm {ln}\left( \frac{m}{l} \right) \left( \frac{l^{\gamma }E_{(l)\, }}{\sum \limits _{s=1}^{C-1} {s^{\gamma }E_{(s)}} } \right) \left( \frac{m^{\gamma }E_{(m)}}{\sum \limits _{s=1}^{C-1} {s^{\gamma }E_{(s)}} } \right) }\\&\quad {\left\{ \ln \left( lm \right) -2\frac{\sum \limits _{s=1}^{C-1} {\ln \left( s \right) s^{\gamma }E_{(s)}} }{\sum \limits _{s=1}^{C-1} {s^{\gamma }E_{(s)}} } \right\} \left\{ \frac{O_{(l)}}{{\, E}_{(l)}}-\frac{O_{(m)}}{{\, E}_{(m)}} \right\} } \, \end{aligned}$$

Rewriting the term in the first set of accolades yields the result, completing the proof. $\square$

Like the first-order derivative in (13), the second-order derivative in (14) is a weighted sum taken over all pairs of different category distances *m* and $l\,{<}\,m$. As reflected by the term $(O_{\left( l \right) }/E_{\left( l \right) })-\mathrm {\, }(O_{\left( m \right) }/E_{\left( m \right) })$, each component compares its smaller distance *l* with its larger distance *m* in terms of the ratio of observed to expected-by-chance frequency. The only difference between the two derivatives is the componentwise multiplier in the first set of accolades in (14). If this multiplier is positive, the component affects the first-order and second-order derivatives in the same direction: The component’s comparison of category distances increasingly shapes the relationship between power parameter $\gamma$ and coefficient $I_{w}$ (i.e., susceptibility tends to intensify) as $\gamma$ increases. Conversely, a negative multiplier implies opposite effects on the two derivatives: The component’s influence reduces (i.e., susceptibility tends to weaken) as $\gamma$ increases. Because the multiplier is increasing in the distances *l* and *m*, components comparing larger category distances become relatively more influential than components comparing smaller category distances as the power parameter increases. Furthermore, settings with more categories (i.e., higher *C*) are more likely to have substantial multipliers, making large *changes* in coefficient susceptibility more likely. The reason is that the term $\mathrm {ln(}lm\mathrm {/}s^{2})$ in the multiplier in (14) can take more extreme values as *C* increases.

## Conditions for Change in Coefficient Susceptibility

A necessary and sufficient condition follows from (13) and (14) in Theorems 1 and 2:

### Corollary 7

As power parameter $\gamma$ *in weighting scheme* (2) *increases, the susceptibility of coefficient* $I_{w}$ in (12) to $\gamma$ intensifies if the first-order derivative $dI_{w}/d\gamma$ in (13) and the second-order derivative $d^{2}I_{w}/{(d\gamma )}^{2}$ in (14) have the same sign and weakens if these two derivatives have opposite signs. Equivalently, as $\gamma$ increases, coefficient susceptibility intensifies if

$$\begin{aligned} \sum \limits _{m=2}^{C-1} \sum \limits _{l<m} {\mathrm {ln}\left( \frac{m}{l} \right) l^{\gamma }m^{\gamma }E_{\left( l \right) }E_{\left( m \right) }\left\{ \frac{O_{\left( l \right) }}{E_{\left( l \right) }}-\frac{O_{\left( m \right) }}{E_{\left( m \right) }} \right\} } \end{aligned}$$

and

$$\begin{aligned} \sum \limits _{m=2}^{C-1} \sum \limits _{l<m} {\mathrm {ln}\!\left( \frac{m}{l} \right) l^{\gamma }m^{\gamma }E_{\left( l \right) }E_{\left( m \right) }\left\{ \sum \limits _{s=1}^{C-1} {\mathrm {ln(}lm\mathrm {/}s^{2}\mathrm {)}s^{\gamma }E_{\left( s \right) }} \right\} \left\{ \frac{O_{\left( l \right) }}{E_{\left( l \right) }}-\frac{O_{\left( m \right) }}{E_{\left( m \right) }} \right\} } \end{aligned}$$

have the same sign and weakens if these two expressions have opposite signs.

### Proof

This follows from the definitions of first-order and second-order derivatives. We obtain the two expressions in the second half of Corollary 7 by ignoring the $\sum \nolimits _{s=1}^{C-1} {s^{\gamma }E_{\left( s \right) }}$ terms that are always positive and do not determine the signs of $dI_{w}/d\gamma$ in (13) and $d^{2}I_{w}/{(d\gamma )}^{2}$ in (14). $\square$

We expect that coefficient susceptibility often weakens as power parameter $\gamma$ increases. The reason is that, especially for high $\gamma$, large category distances *s* correspond to *both* high values of $s^{\gamma }$ and negative values of $\mathrm {ln(}lm\mathrm {/}s^{2}\mathrm {)}$ in $\sum \nolimits _{s=1}^{C-1} {\mathrm {ln(}lm\mathrm {/}s^{2}\mathrm {)}s^{\gamma }E_{\left( s \right) }}$, whereas small category distances *s* correspond to *both* low values of $s^{\gamma }$ and positive values of $\mathrm {ln(}lm\mathrm {/}s^{2}\mathrm {)}$. Thus, large distances *s* tend to make large negative contributions to $\sum \nolimits _{s=1}^{C-1} {\mathrm {ln(}lm\mathrm {/}s^{2}\mathrm {)}s^{\gamma }E_{\left( s \right) }}$, whereas small distances *s* tend to make only small positive contributions, triggering opposite signs in Corollary 7. As this mechanism for weakening coefficient susceptibility becomes increasingly strong for higher values of the power parameter, coefficient susceptibility ultimately converges to zero. Furthermore, the mechanism is more prominent if the values of $E_{\left( s \right) }$ remain substantial for large *s*,  so large category distances are relatively likely to occur by chance.

For $$C=3$$ categories (with the only feasible combination being $$l=1$$ and $$m=2)$$, we can write the second-order derivative in (14) as a multiple of the first-order derivative in (13):

15 $$\begin{aligned} \left. \frac{d^{2}I_{w}}{{(d\gamma )}^{2}} \right| _{C=3}\!=\!\frac{\ln \left( 2 \right) E_{(1)}\!+\!\mathrm {ln}(1/2) 2^{\gamma }E_{\left( 2 \right) }}{E_{(1)}\!+\!2^{\gamma }E_{(2)}}\times \left. \frac{dI_{w}}{d\gamma } \right| _{C=3}\!=\!\frac{\mathrm {ln}\left( 2 \right) \{E_{\left( 1 \right) }-2^{\gamma }E_{\left( 2 \right) }\}}{E_{(1)}+2^{\gamma }E_{(2)}}\times \left. \frac{dI_{w}}{d\gamma } \right| _{C=3},\,\nonumber \\ \end{aligned}$$

where the fraction in (15) is the multiplier in (14) for $l=1$ and $m=2$. Equivalently,

16 $$\begin{aligned} {\left. \frac{d^{2}I_{w}}{{(d\gamma )}^{2}} \right| _{C=3}\, }\Big /{\, \left. \frac{dI_{w}}{d\gamma } \right| _{C=3}}=\frac{\mathrm {ln}\left( 2 \right) \{E_{\left( 1 \right) }-2^{\gamma }E_{\left( 2 \right) }\}}{E_{(1)}+2^{\gamma }E_{(2)}}.\, \end{aligned}$$

We obtain the following result for three-category settings, where the relationship between the power parameter and the coefficient value is monotonic due to Corollary 2:

### Corollary 8

As power parameter $\gamma$ in weighting scheme (2) increases in settings with three categories, the chance-corrected weighted agreement coefficient $I_{w}$ in (12) becomes more susceptible to $\gamma$ until $\gamma ^{*}=\mathrm {ln(}E_{\left( 1 \right) }/E_{\left( 2 \right) })/\mathrm {ln}(2)$ . Next, $I_{w}$ becomes less susceptible.

### Proof

Equation (15) implies that the second-order derivative has the same sign as the first-order derivative if $E_{(1)}-2^{\gamma }E_{\left( 2 \right) }>0$, or equivalently if $\gamma <\mathrm {ln(}E_{(1)}/E_{(2)})/\mathrm {ln}(2)$. Similarly, the signs of the first-order and second-order derivatives are opposite if $\gamma >\mathrm {ln(}E_{(1)}/E_{(2)})/\mathrm {ln}(2).$ So, coefficient susceptibility intensifies until $\gamma ^{*}=\mathrm {ln(}E_{(1)}/E_{(2)})/\mathrm {ln}(2)$ and weakens after. $\square$

Thus, if $C=3$, there is a value of the power parameter for which the chance-corrected weighted agreement coefficient $I_{w}$ is most susceptible to this parameter, and this value $\gamma ^{*}$ is easy to compute. Furthermore, the weighted *S*-coefficient is most susceptible to linear weights:

### Corollary 9

As power parameter $\gamma$ in weighting scheme (2) increases in settings with three categories, the weighted S-coefficient becomes more susceptible to $\gamma$ until $\gamma ^{*}=1$. Next, the weighted S-coefficient becomes less susceptible.

### Proof

Because $E_{\left( l \right) }=2(C-l)/C^{2}$, the weighted $S-$coefficient with $C=3$ categories implies that $E_{(1)}=4/9$ and $E_{(2)}=2/9$. Substituting into Corollary 8 yields $\gamma ^{*}=\mathrm {ln(}2)/\mathrm {ln}(2)=1$. $\square$

**Fig. 1:**
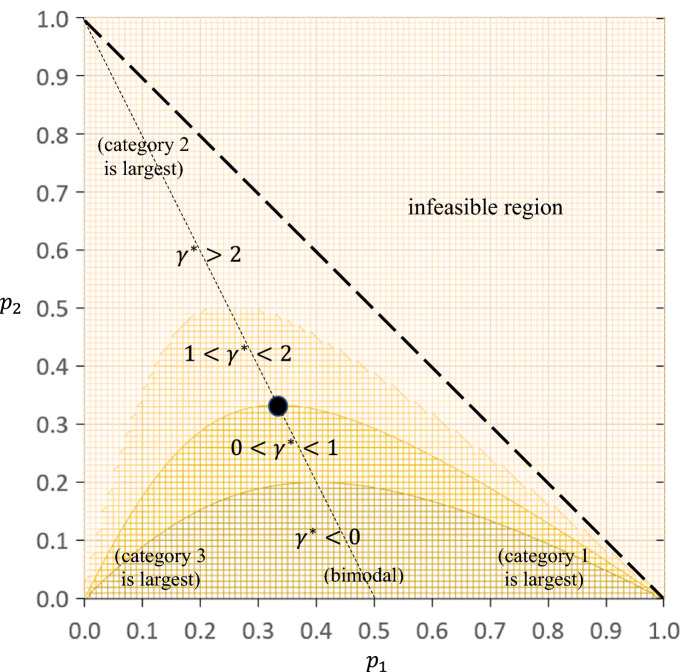
Contour Plot Showing the Value of the Power Parameter Corresponding to Maximum Coefficient Susceptibility ($$\gamma ^{*})$$ for Varying Category Proportions ($$p_{1}$$ and $$p_{2})$$ in Three-Category Settings. *Note.* The dotted line captures all symmetric distributions for the category proportions (i.e., $$p_{1}=p_{3}$$, where $$p_{3}=1-p_{1}-p_{2})$$; the dot is the weighted *S*-coefficient (i.e., $$p_{1}=p_{2}=p_{3}=1/3)$$.

The contour plot in Figure 1 visualizes Corollary 8 for all settings with three categories and $p_{c}=q_{c}$, $c=1,\ldots ,C$; it visualizes Corollary 9 for the weighted *S*-coefficient by showing a dot at $p_{1}=p_{2}=1/3$, where $\gamma ^{*}=1$. The figure shows a wide range of possible values for $\gamma ^{*}$. Coefficient susceptibility weakens monotonically as the power parameter increases (i.e., $\gamma ^{*}<0)$ if the distribution of category proportions is substantially bimodal (in the bottom part of the figure, triggering high $E_{(s)}$ for large *s* in Corollary 7). Conversely, coefficient susceptibility intensifies monotonically at least up to quadratic weights (i.e., $\gamma ^{*}>2)$ if the distribution is strongly unimodal, entailing a middle category that is substantially larger than the smallest corner category (in the top, left, and right parts of the figure, triggering low $E_{\left( s \right) }$ for large *s* in Corollary 7). Furthermore, coefficient susceptibility is most extreme between identity and quadratic weights (i.e., $0<\gamma ^{*}<2)$ if the category proportions $p_{1}$, $p_{2}$, and $p_{3}$ are somewhat balanced. We note that small changes in the distribution of category proportions may substantially affect the value of $\gamma ^{*}$ if one of the corner categories strongly dominates (in the figure’s bottom left and right parts).

## Descriptive Measures of Coefficient Susceptibility

Based on the preceding analysis, we propose descriptive measures that summarize various aspects of coefficient susceptibility for any data set with rater-based classifications. As shorthand notation, we denote the first-order derivative by $D_{1}(\gamma )$ and the second-order derivative by $D_{2}(\gamma )$.

First, researchers may use the first-order derivative $D_{1}(\gamma )$ to describe how the coefficient value reacts to changes in the chosen value of power parameter $\gamma$. The sign of $D_{1}\left( \gamma \right)$ reveals the direction of dependence; the absolute value quantifies the degree of coefficient susceptibility. The measure $D_{1}(\gamma )$ is the change in the value of coefficient $I_{w}$ in response to a small change in $\gamma$, expressed as a multiple of this change in $\gamma$. This measure of coefficient susceptibility is invariant to the amount of curvature that is present in the relationship between $\gamma$ and $I_{w}$. Interpretation is most straightforward for settings in which the relationship between $\gamma$ and $I_{w}$ is (almost) linear. For example, $D_{1}\!\left( \gamma \right) =.10$ would mean that the value of $I_{w}$ changes by (approximately) .10 if $\gamma$ changes by one point. Settings with substantial curvature in the relationship between $\gamma$ and $I_{w}$ require combining $D_{1}\!\left( \gamma \right)$ with a measure of curvature, that is, *change* in coefficient susceptibility as $\gamma$ changes (as discussed below). In settings with three categories, researchers may also report $D_{1}(\gamma ^{*})$, that is, the first-order derivative evaluated at the value of the power parameter for which coefficient $I_{w}$ is most susceptible, where Corollary 8 defines $\gamma ^{*}$. This measure provides a tight upper bound for the degree of coefficient susceptibility over the entire range of $\gamma$; it is independent of the chosen value of the power parameter.

Second, researchers may use the ratio $D_{2}(\gamma )/D_{1}(\gamma )$ to describe the amount of curvature that is present in the relationship between power parameter $\gamma$ and coefficient $I_{w}$, or equivalently, to describe the *change* in coefficient susceptibility as $\gamma$ changes (Pratt 1964). A positive sign of $D_{2}(\gamma )/D_{1}(\gamma )$ indicates that the coefficient value changes more when $\gamma$ increases than when $\gamma$ decreases (i.e., susceptibility intensifies as $\gamma$ increases), whereas a negative sign indicates the opposite (i.e., susceptibility weakens as $\gamma$ increases). The absolute value of $D_{2}(\gamma )/D_{1}(\gamma )$ quantifies the change in coefficient susceptibility as a fraction of the amount of susceptibility that is present. Thus, $D_{2}(\gamma )/D_{1}(\gamma )$ is a scaled measure that is invariant to the actual degree of susceptibility. For settings with three categories, this ratio measure reduces to (16), a simple closed-form expression. We note that $D_{1}(\gamma )$ and $D_{2}(\gamma )/D_{1}(\gamma )$ complement each other: The former describes coefficient susceptibility independent of the amount of curvature in the relationship between $\gamma$ and $I_{w}$; the latter describes curvature (or change in susceptibility) independent of the amount of susceptibility.

We illustrate the measures $D_{1}(\gamma )$ and $D_{2}(\gamma )/D_{1}(\gamma )$ for linear weights by considering 31 data sets from the literature. Contingent on our library access, these data sets originate from two literature reviews by Warrens (2013) and Warrens (2014), supplemented by other data sets that we obtained by checking lists of references and additional well-known studies of interrater agreement. In addition to $D_{1}(\gamma )$ and $D_{2}(\gamma )/D_{1}(\gamma )$, we show $D_{1}(\gamma ^{*})$ and the corresponding value of $\gamma ^{*}$ for all data sets with $C=3$ categories. For settings with $R=2$ raters, we implement Cohen’s kappa, which is the most frequently used coefficient in practice. For settings with $R\ge 3$ raters, we implement Fleiss’ kappa, proposed in the literature as an easy generalization of Cohen’s kappa beyond two raters (although it generalizes Scott’s pi rather than Cohen’s kappa).

**Table 2:** Measures of coefficient susceptibility and their interpretation for 31 data sets from literature.

Source	*R*	*C*	$I_{w}$	$D_{1}(\gamma )$	$D_{1}(\gamma ^{*})\,$	$\gamma ^{*}$	$D_{2}/D_{1}$	Degree	Direction	Change
Cohen (1960)	2	3	.023	.118	.118	.869	−.032	Very high	Positive	Minor
Cohen (1960)	2	3	.474	−.019	−.019	1.305	.073	Moderate	Negative	Minor
Fleiss (1971)	2	3	.128	−.023	−.024	.441	−.133	Moderate	Negative	Weakening
Fleiss (1971)	2	3	.250	.043	.043	1.131	.032	Moderate	Positive	Minor
Guggenmoos-Holzmann and Vonk (1998)	2	3	.737	.009	.013	2.776	.380	Moderate	Positive	Intensifying
Spitzer and Fleiss (1974)	2	3	.722	.040	.052	−.515	−.334	Moderate	Positive	Weakening
Sim and Wright (2005)	2	3	.523	.068	.071	1.545	.129	High	Positive	Intensifying
Sim and Wright (2005)	2	4	.612	.065	-	-	−.098	High	Positive	Minor
De Raadt et al. (2021)	2	4	.677	.085	-	-	.260	High	Positive	Intensifying
Hand et al. (1994, p.170)	2	4	.380	.165	-	-	−.181	Very high	Positive	Weakening
Hand et al. (1994, p.170)	2	4	.619	.163	-	-	−.378	Very high	Positive	Weakening
Hand et al. (1994, p.170)	2	4	.768	.160	-	-	−.412	Very high	Positive	Weakening
Landis and Koch (1977)	2	4	.380	.165	-	-	−.181	Very high	Positive	Weakening
Landis and Koch (1977)	2	4	.477	.172	-	-	−.206	Very high	Positive	Weakening
Simonoff (2003, p.288)	2	4	.790	.113	-	-	−.183	Very high	Positive	Weakening
Simonoff (2003, p.303)	2	4	.307	.201	-	-	−.054	Very high	Positive	Minor
Simonoff (2003, p.303)	2	4	.575	.031	-	-	.011	Moderate	Positive	Minor
Agresti (1988)	2	5	.649	.146	-	-	−.161	Very high	Positive	Weakening
Graham and Jackson (1993)	2	5	.665	.151	-	-	−.288	Very high	Positive	Weakening
Maria and Victorino (1997)	2	5	.805	.056	-	-	.142	High	Positive	Intensifying
Maria and Victorino (1997)	2	5	.846	.088	-	-	−.149	High	Positive	Weakening
Simonoff (2003, p.272)	2	5	.944	.028	-	-	−.264	Moderate	Positive	Weakening
Van Swieten et al. (1988)	2	6	.780	.174	-	-	−.561	Very high	Positive	Weakening
Cookson et al. (1986)	2	8	.475	.150	-	-	.006	Very high	Positive	Minor
Maclure and Willett (1987)	2	12	.478	.265	-	-	−.747	Very high	Positive	Weakening
Tinsley and Weiss (1975)	3	3	.108	−.082	−.100	−.320	−.297	High	Negative	Weakening
Gwet (2014, p.96)	3	5	.277	.135	-	-	.191	Very high	Positive	Intensifying
Gwet (2014 p.370)	3	5	.237	.234	-	-	−.019	Very high	Positive	Minor
Gwet (2014 p.372)	4	5	.591	.163	-	-	−.234	Very high	Positive	Weakening
Fleiss et al. (2003, p.615)	5	3	.320	−.094	−.099	.341	−.156	High	Negative	Weakening
Holmquist et al. (1967)	7	5	.510	.150	-	-	−.169	Very high	Positive	Weakening

Linear weights ($\gamma =1)$, Cohen’s kappa if $R=2$ raters, and Fleiss’ kappa if $R\ge 3$ raters; degree of coefficient susceptibility is considered moderate if $\left| D_{1} \right| <.05$, high if $.05\le \left| D_{1} \right| <.10$, and very high if $\left| D_{1} \right| \ge .10$; change in susceptibility (i.e., curvature) is considered minor if $\left| D_{2}/D_{1} \right| <.10$; $D_{1}(\gamma ^{*})\,$and $\gamma ^{*}$ exist only in settings with $C=3$ categories (see Corollary 8).

We provide Ox source code as online supplementary material on the journal’s website. Ox is free of charge for academics, and downloads are available at doornik.com/download.html (Doornik 2007). The default value of the power parameter in the Ox source code is $\gamma =1$, but users can easily adjust its value. To compute the measures $D_{1}(\gamma )$, $D_{2}(\gamma )/D_{1}(\gamma )$, $D_{1}(\gamma ^{*})$, and $\gamma ^{*}$, we recommend editing the first data set in the Ox source code if users wish to implement Cohen’s kappa to analyze a data set in $C\times C$ contingency table format. Alternatively, we recommend editing the last data set in the Ox source code if the data set is an $N\times C$ table containing the rater frequencies $R_{i,c}$. In the latter case, the calculations assume that the coefficient is Fleiss’ kappa, which coincides with Scott’s pi if there are $R=2$ raters. Users can run the source code in the Ox editor by first clicking on “Modules” and next clicking on “Ox.” This automatically prints all computed statistics.

Table 2 shows that $$D_{1}\!\!\left( \gamma \right) >0$$ for 27 out of 31 data sets, confirming that coefficient values usually increase as the power parameter of the weighting scheme increases. Furthermore, the degree of coefficient susceptibility is often high: $$\left| D_{1}\left( \gamma \right) \right| \ge .10$$ for 17 data sets, $$.05\le \left| D_{1}\left( \gamma \right) \right| <.10$$ for 7 data sets, and $$\left| D_{1}\left( \gamma \right) \right| <.05$$ for only 7 data sets. For the data sets with $$C=3$$ categories (implying monotonicity), the values of $$\gamma ^{*}$$ vary substantially, confirming that the point until which coefficient susceptibility intensifies depends on the specific data set. As anticipated, the number of categories *C* correlates strongly with the degree of coefficient susceptibility $$\left| D_{1}\left( \gamma \right) \right| $$, with a correlation coefficient of .59. Furthermore, the values of $$D_{2}\left( \gamma \right) /D_{1}\left( \gamma \right) $$ show that coefficient susceptibility often weakens as the power parameter increases (18 data sets), although it sometimes intensifies (5 data sets) or is almost constant (8 data sets). As anticipated, substantial change in coefficient susceptibility (i.e., curvature in the relationship between $$\gamma $$ and $$I_{w})$$ occurs most often when the number of categories *C* is high, with a correlation coefficient of .48.

## Coefficient Values for Unreported Values of Power Parameter

Beyond interpretation of coefficient susceptibility in terms of positive or negative and intensifying or weakening, the summary measures $D_{1}\left( \gamma \right)$ and $D_{2}\left( \gamma \right) /D_{1}\left( \gamma \right)$ help researchers obtain (approximate) coefficient values for unreported values of the power parameter. For example, Table 2 shows the results for linear weights only. Still, we can use these results to estimate the coefficient values for other choices, such as identity, radical, or quadratic weights. Furthermore, these estimates do not require access to the original data because the coefficient $I_{w}$ computed at $\gamma$, the first-order derivative $D_{1}\left( \gamma \right)$, and the ratio $D_{2}\left( \gamma \right) /D_{1}\left( \gamma \right)$ are sufficient statistics. The second-order Taylor series (i.e., quadratic) approximation of coefficient $I_{w}$, computed for an alternative power parameter value $\gamma +\mathrm {\Delta }\gamma$, is given by

17 $$\begin{aligned} I_{w}\left( \gamma +\mathrm {\Delta }\gamma \right) \approx I_{w}\left( \gamma \right) +D_{1}\left( \gamma \right) \left( \mathrm {\Delta }\gamma +\frac{1}{2}\frac{D_{2}\left( \gamma \right) }{D_{1}\left( \gamma \right) }\left( \mathrm {\Delta }\gamma \right) ^{2} \right) ,\, \end{aligned}$$

where the right-hand side is the heuristic value, and $\mathrm {\Delta }\gamma$ is the change in the power parameter.

Table 3 shows the deviations between the actual value of $I_{w}\left( \gamma +\mathrm {\Delta }\gamma \right)$ and the corresponding heuristic value in (17) for all 31 data sets in Table 2. We consider $\gamma =1$ and $\gamma =2$ for the original value of the power parameter (i.e., linear and quadratic weights). Next, we change the value of $\gamma$: $\mathrm {\Delta }\gamma =-1$, $\mathrm {\Delta }\gamma =-.5$, $\mathrm {\Delta }\gamma =.5$, and $\mathrm {\Delta }\gamma =1$, resulting in $2\times 4=8$ different scenarios. The heuristic is generally accurate. The mean absolute deviation based on all 31 $\times$ 8 cells in Table 3 is .002, and the corresponding mean absolute *percent* deviation is .99, approximately one percent. More precisely, the mean absolute deviation across the 31 data sets is .005 or less for *each* of the eight scenarios, and this deviation is .001 or less for the four scenarios with either $\mathrm {\Delta }\gamma =-.5$ or $\mathrm {\Delta }\gamma =.5$. Similarly, the *maximum* absolute deviation across 30 of the 31 data sets is .011 or less for *each* of the eight scenarios. Furthermore, the absolute deviation remains modest for the excluded data set from Maclure and Willett (1987), with $C=12$ categories and extreme levels of susceptibility and curvature. This deviation is .019 for $\gamma =1$ and $\mathrm {\Delta }\gamma =1$, .016 for $\gamma =2$ and $\mathrm {\Delta }\gamma =-1$, and .011 or less for the other six scenarios.

**Table 3:** Deviation between second-order Taylor series heuristic and actual coefficient value for different original values of power parameter ($\mathrm {\gamma })$ and changes ($\mathrm {\Delta \gamma )}$.

$\gamma$ (original value)			1.0	1.0	1.0	1.0	2.0	2.0	2.0	2.0
$\mathrm {\Delta }\gamma$ (change)	*R*	*C*	$-$ 1.0	$-$ .5	.5	1.0	$-$ 1.0	−.5	.5	1.0
Cohen (1960)	2	3	$-$.005	$-$ .001	.001	.004	$-$.003	$-$.000	.000	.002
Cohen (1960)	2	3	.001	.000	$-$ .000	$-$.001	.001	.000	$-$.000	$-$.000
Fleiss (1971)	2	3	.001	.000	$-$ .000	$-$.001	.000	.000	$-$.000	$-$.000
Fleiss (1971)	2	3	$-$.002	$-$ .000	.000	.002	$-$.001	$-$.000	.000	.001
Guggenmoos-Holzmann and Vonk (1998)	2	3	.000	$-$ .000	.000	.000	$-$.000	$-$.000	.000	.000
Spitzer and Fleiss (1974)	2	3	$-$.001	$-$ .000	.000	.000	.000	.000	$-$.000	$-$.001
Sim and Wright (2005)	2	3	$-$.002	$-$ .000	.000	.003	$-$.003	$-$.000	.000	.002
Sim and Wright (2005)	2	4	$-$.003	$-$ .000	.000	.002	$-$.001	$-$.000	.000	.000
De Raadt et al. (2021)	2	4	$-$.002	$-$ .000	.000	.003	$-$.004	$-$.001	.001	.004
Hand et al. (1994, p.170)	2	4	$-$.008	$-$ .001	.001	.005	$-$.002	$-$.000	$-$.000	$-$.001
Hand et al. (1994, p.170)	2	4	−.007	$-$ .001	.000	.002	.002	.000	$-$.000	$-$.004
Hand et al. (1994, p.170)	2	4	$-$.007	$-$ .001	.000	.002	.003	.000	$-$.001	$-$.004
Landis and Koch (1977)	2	4	−.008	$-$ .001	.001	.005	−.002	−.000	−.000	−.001
Landis and Koch (1977)	2	4	−.008	$-$ .001	.001	.006	−.002	−.000	.000	−.001
Simonoff (2003, p.288)	2	4	−.006	$-$ .001	.001	.004	−.002	−.000	−.000	−.001
Simonoff (2003, p.303)	2	4	−.009	$-$ .001	.001	.008	−.005	−.000	.000	.001
Simonoff (2003, p.303)	2	4	−.002	$-$ .000	.000	.003	−.003	−.000	.000	.002
Agresti (1988)	2	5	−.007	$-$ .001	.001	.005	−.002	−.000	−.000	−.001
Graham and Jackson (1993)	2	5	−.007	$-$ .001	.000	.003	.001	.000	−.000	−.003
Maria and Victorino (1997)	2	5	−.002	$-$ .000	.000	.003	−.003	−.000	.000	.002
Maria and Victorino (1997)	2	5	−.006	$-$ .001	.001	.005	−.002	−.000	−.000	−.001
Simonoff (2003, p.272)	2	5	−.001	$-$ .000	.000	.001	−.000	.000	−.000	−.000
Van Swieten et al. (1988)	2	6	−.006	$-$ .000	$-$ .000	−.002	.006	.001	−.001	−.007
Cookson et al. (1986)	2	8	−.005	$-$ .001	.001	.005	−.003	−.000	.000	.002
Maclure and Willett (1987)	2	12	.010	.002	$-$ .002	−.019	.016	.002	−.002	−.011
Tinsley and Weiss (1975)	3	3	.002	.000	$-$ .000	−.001	−.000	−.000	.000	.001
Gwet (2014 p.96)	3	5	−.005	$-$ .001	.001	.006	−.005	−.001	.001	.004
Gwet (2014 p.370)	3	5	−.011	$-$ .001	.001	.010	−.006	−.001	.000	.002
Gwet (2014 p.372)	4	5	−.002	$-$ .000	.000	.001	−.000	.000	−.000	−.001
Fleiss et al. (2003, p.615)	5	3	.004	.000	$-$ .000	−.003	.001	.000	−.000	−.000
Holmquist et al. (1967)	7	5	−.007	$-$ .001	.001	.005	−.002	−.000	−.000	−.001

Cohen’s kappa if $R=2$ raters, and Fleiss’ kappa if $R\ge 3$ raters.

## Example: Two Raters and Three Categories

We consider a contingency table from Cohen (1960) that corresponds to the first row of Table 2. Table 4 reproduces the observed and expected fractions of items for which the choices by the two raters result in the corresponding category combination. As there are three categories, the maximum possible category distance (i.e., distance to the main diagonal) is two. For Cohen’s kappa with three categories, Corollaries 1, 2, 3, 7, and 8 apply (see Table 1).

The observed fraction of items with category distance one equals $O_{(1)}=.12+.15+.13+.16=.56$; the corresponding fraction expected by chance is $E_{(1)}=.12+.06+.15+.09=.42$, implying that the ratio of observed to expected-by chance for category distance $l=1$ becomes $O_{\left( 1 \right) }/E_{\left( 1 \right) }=.56/.42=1.333$. Similarly, the observed fraction of items with category distance two equals $O_{(2)}=.03+.12=.15$, and the corresponding fraction expected by chance is $E_{(2)}=.08+.15=.23$. Thus, the ratio of observed to expected-by-chance for category distance $l=2$ becomes $O_{\left( 2 \right) }/E_{\left( 2 \right) }=.15/.23=.652$. Because the ratio of observed to expected by chance is higher for category distance one (i.e., closer to the main diagonal) than for category distance two (i.e., farther away), coefficient $I_{w}$ increases monotonically as power parameter $\gamma$ increases (Corollaries 2 and 3). Furthermore, coefficient $I_{w}$ is most susceptible to $\gamma$ at $\gamma ^{*}=\mathrm {ln}(E_{\left( 1 \right) }/E_{\left( 2 \right) })/\mathrm {ln}(2)=\mathrm {ln}(.42/.23)/\mathrm {ln}(2)=.869$, close to linear weights (Corollary 8).

Because the only feasible pair of category distances for $C=3$ is $l=1$ and $m=2$, it follows from (13) that the first-order derivative for linear weights (i.e., $\gamma =1)$ becomes

$$\begin{aligned} D_{1}(\gamma )= & {} \mathrm {ln}\left( \frac{m}{l} \right) \left( \frac{l^{\gamma }E_{\left( l \right) }}{\sum \limits _{s=1}^{C-1} {s^{\gamma }E_{\left( s \right) }} } \right) \left( \frac{m^{\gamma }E_{\left( m \right) }}{\sum \limits _{s=1}^{C-1} {s^{\gamma }E_{\left( s \right) }} } \right) \left\{ \frac{O_{\left( l \right) }}{E_{\left( l \right) }}-\frac{O_{\left( m \right) }}{E_{\left( m \right) }} \right\} \\= & {} \mathrm {ln}\left( \frac{2}{1} \right) \left( \frac{1\times .42}{1\times .42+2\times .23} \right) \left( \frac{2\times .23}{1\times .42+2\times .23} \right) \left\{ 1.333-.652 \right\} =.118. \end{aligned}$$

Thus, the coefficient value reacts strongly to changes in the power parameter, and there is indeed a positive relationship (Corollary 1). Furthermore, $D_{1}\left( \gamma ^{*} \right) =.118$ at $\gamma ^{*}=.869$, so coefficient susceptibility for linear weights is essentially the same as the maximum over the entire range of $\gamma$. Next, we use (16) to compute the ratio of derivatives:

$$\begin{aligned} {D_{2}(\gamma )}\Big /{D_{1}(\gamma )}=\frac{\mathrm {ln}\left( 2 \right) \{E_{\left( 1 \right) }-2^{\gamma }E_{\left( 2 \right) }\}}{E_{(1)}+2^{\gamma }E_{(2)}}=\frac{\ln \left( 2 \right) \{.42-2\times .23\}}{.42+2\times .23}=-.032. \end{aligned}$$

As the derivatives $D_{1}(\gamma )$ and $D_{2}(\gamma )$ have opposite signs, coefficient susceptibility weakens as the power parameter increases (Corollary 7). However, ${D_{2}(\gamma )}/{D_{1}(\gamma )}\approx 0$, implying almost linearity in the relationship between the power parameter and the coefficient value.

Using (12) and Table 4, we compute the coefficient value for linear weights:

$$\begin{aligned} I_{w}\left( \gamma \right) =1-\frac{\sum \limits _{l=1}^{C-1} {l^{\gamma }O_{\left( l \right) }} }{\sum \limits _{l=1}^{C-1} {l^{\gamma }E_{\left( l \right) }} } =1-\frac{1\times .56+2\times ..15}{1\times .42+2\times .23}=1-\frac{.86}{.88}=.023. \end{aligned}$$

When moving from linear to identity weights (i.e., $\gamma =1$ and $\mathrm {\Delta }\gamma =-1)$, the heuristic in (17) is

$$\begin{aligned} I_{w}\left( \gamma \right) +D_{1}\left( \gamma \right) \left( \mathrm {\Delta }\gamma +\frac{1}{2}\frac{D_{2}\left( \gamma \right) }{D_{1}\left( \gamma \right) }\left( \mathrm {\Delta }\gamma \right) ^{2} \right)&=.023+.118\\&\quad \times \left( -1+\frac{1}{2}\times -.032\times {(-1)}^{2} \right) =-.097. \end{aligned}$$

Because the actual value of Cohen’s (unweighted) kappa is −.092, the corresponding deviation becomes $-.097-\left( -.092 \right) =-.005$, as shown in the first cell of Table 3. Similarly, when moving from identity to quadratic weights (i.e., $\gamma =1$ and $\mathrm {\Delta }\gamma =1)$, the heuristic in (17) yields

$$\begin{aligned} I_{w}\!\left( \gamma \right) +\!D_{1}\left( \gamma \right) \left( \mathrm {\Delta }\gamma +\frac{1}{2}\frac{D_{2}\left( \gamma \right) }{D_{1}\left( \gamma \right) }\left( \mathrm {\Delta }\gamma \right) ^{2} \right) =.023+.118\times \left( 1+\frac{1}{2}\times -.032\times {(1)}^{2} \right) =.139. \end{aligned}$$

Cohen’s weighted kappa with quadratic weights equals .134, which is again quite close.

**Table 4:** Calculations in example with two raters and three categories.

Observed	.25	.13	.12	**.50**
	.12	.02	.16	**.30**
	.03	.15	.02	**.20**
	**.40**	**.30**	**.30**	**1.00**
Expected	.20	.15	.15	**.50**
	.12	.09	.09	**.30**
	.08	.06	.06	**.20**
	**.40**	**.30**	**.30**	**1.00**
Distance	$l\,=\,$ 1	$l\,=\,$ 2		
$O_{(l)}$	.56	.15		
$E_{(l)}$	.42	.23		
$O_{(l)}/E_{(l)}$	1.333	.652		
${(O}_{\left( 1 \right) }/E_{\left( 1 \right) })-{(O}_{\left( 2 \right) }/E_{\left( 2 \right) })$			.681	
$\gamma ^{*}=\mathrm {ln(}E_{(1)}/E_{(2)})/\mathrm {ln}(2)$			.869	
For$\, \gamma =1$				
$D_{1}(\gamma )$			.118	
${D_{2}(\gamma )}/{D_{1}(\gamma )}$			−.032	
$I_{w}(\gamma )$			.023	
Actual and heuristic values of $I_{w}$ for other $\gamma$				
$\, \gamma =0$		−.092	−.097	
$\, \gamma =2$		.134	.139	

## Example: Four Raters and Five Categories

We consider a data set from Gwet (2014, p.372), included in Table 2. As there are five categories, the maximum possible category distance is four. As we consider Fleiss’ kappa with more than three categories, Corollaries 1, 3, and 7 apply (see Table 1). The top part of Table 5 shows the observed and expected fractions of cases (i.e., combinations of items and rater pairs) for the four distances $l=1,\ldots ,4$, together with the corresponding ratios $O_{(l)}/E_{(l)}$. Because $O_{(l)}/E_{(l)}$ is decreasing in the category distance *l*, coefficient $I_{w}$ increases monotonically as power parameter $\gamma$ increases (Corollary 3).

**Table 5:** Calculations in example with four raters and five categories.

Distance	$l\,=\,$ 1	$l\,=\,$ 2	$l \,=\,$ 3	$l \,=\,$ 4
$O_{(l)}$	.155	.253	.011	.000
$\, E_{(l)}$	.110	.378	.062	.162
${\, O}_{(l)}/E_{(l)}$	1.409	.670	.187	.000
$${\, (O}_{\left( l \right) }/E_{\left( l \right) })-{(O}_{\left( m \right) }/E_{\left( m \right) })$$				
		$l=1$	$l=2$	$l=3$
$m=2\,$		.739		
$m=3\,$		1.222	.483	
$m=4\,$		1.409	.670	.187
Contributions to $dI_{w}/d\gamma$ for $\gamma =1$				
		$l=1$	$l=2$	$l=3$
$m=2\,$		.015		
$m=3\,$		.009	.009	
$m=4\,$		.048	.079	.002
For$\, \gamma =1$				
$\, D_{1}(\gamma )$			.163	
$\, {D_{2}(\gamma )}/{D_{1}(\gamma )}$			−.234	
$\, I_{w}(\gamma )$			.591	
Actual and heuristic values of $I_{w}$ for other $\gamma$				
$\gamma =0$		.410	.409	
$\gamma =2$		.734	.735	

We compute the differences $(O_{\left( l \right) }/E_{\left( l \right) })-(O_{\left( m \right) }/E_{\left( m \right) })$ for all pairs of different category distances, $m=2,\ldots ,4$, and $l<m$, resulting in six pairwise comparisons of distances. Next, we compute the components of the first-order derivative in (13) for linear weights and these six pairs of distances. For example, the first component, with $l=1$ and $m=2$, is

$$\begin{aligned}&\mathrm {ln}\left( \frac{m}{l} \right) \left( \frac{l^{\gamma }E_{\left( l \right) }}{\sum \limits _{s=1}^{C-1} {s^{\gamma }E_{\left( s \right) }} } \right) \left( \frac{m^{\gamma }E_{\left( m \right) }}{\sum \limits _{s=1}^{C-1} {s^{\gamma }E_{\left( s \right) }} } \right) \left\{ \frac{O_{\left( l \right) }}{E_{\left( l \right) }}-\frac{O_{\left( m \right) }}{E_{\left( m \right) }} \right\} \\&\quad =\mathrm {ln}\left( \frac{2}{1} \right) \left( \frac{1\times .110}{1.700} \right) \times \left( \frac{2\times .378}{1.700} \right) \times .739\\&\quad =.015, \end{aligned}$$

where

$$\begin{aligned} \sum \limits _{s=1}^{C-1} {s^{\gamma }E_{\left( s \right) }} =1\times .110+2\times .378+3\times .062+4\times .162=1.700. \end{aligned}$$

The first-order derivative is the sum of the six components: $D_{1}(\gamma )=.015+.009+.009+.048+.079+.002=.163$. Thus, the relationship between the power parameter and the coefficient value is indeed positive (Corollary 1). As shown in the middle part of Table 5, the coefficient’s strong susceptibility to the power parameter is mainly due to comparisons involving the maximum possible category distance $m=4$ that never actually occurred in the data (i.e., $O_{(4)}/E_{(4)}=.000)$. In particular, the comparisons of the two category distances $l=1$ and $l=2$ with distance $m=4$ (implying large distance ratios *m*/*l* in (13)) contribute substantially to $D_{1}(\gamma )$; they account for more than 80 percent of the total. The remaining distance $l=3$ is unlikely to occur by chance and therefore plays only a minor role: $E_{(3)}=.062$. Using a similar decomposition for the second-order derivative in (14), we obtain $D_{2}(\gamma )/D_{1}(\gamma )=-.234$. As the two derivatives $D_{1}(\gamma )$ and $D_{2}(\gamma )$ have opposite signs, coefficient susceptibility weakens as the power parameter increases (Corollary 7).

Using (12) and Table 5, we compute the coefficient value for linear weights:

$$\begin{aligned} I_{w}\left( \gamma \right)= & {} 1-\frac{\sum \limits _{l=1}^{C-1} {l^{\gamma }O_{\left( l \right) }} }{\sum \limits _{l=1}^{C-1} {l^{\gamma }E_{\left( l \right) }} }=1-\frac{1\times .155+2\times .253+3\times .011+4\times .000}{1\times .110+2\times .378+3\times .062+4\times .162}=1-\frac{.694}{1.700}\\= & {} .591. \end{aligned}$$

As before, we use the second-order Taylor series heuristic in (17) to obtain estimates of $I_{w}$ for identity and quadratic weights, based on the computed measures for linear weights. Gwet (2014, p.150) reported that Fleiss’ kappa with identity weights equals .410 for the considered data set. The heuristic in (17) yields essentially the same coefficient value when moving from linear to identity weights (i.e., $\gamma =1$ and $\mathrm {\Delta }\gamma =-1)$:

$$\begin{aligned} I_{w}\left( \gamma \right) +D_{1}\left( \gamma \right) \left( \mathrm {\Delta }\gamma +\frac{1}{2}\frac{D_{2}\left( \gamma \right) }{D_{1}\left( \gamma \right) }\left( \mathrm {\Delta }\gamma \right) ^{2} \right)= & {} .591+.163\times \left( -1+\frac{1}{2}\times -.234\times {(-1)}^{2} \right) \\ {}= & {} .409. \end{aligned}$$

Similarly, Gwet (2014, p.150) reported that Fleiss’ kappa with quadratic weights equals .734. When moving from linear to quadratic weights (i.e., $\gamma =1$ and $\mathrm {\Delta }\gamma =1)$, the heuristic yields

$$\begin{aligned} I_{w}\left( \gamma \right) +D_{1}\left( \gamma \right) \left( \mathrm {\Delta }\gamma +\frac{1}{2}\frac{D_{2}\left( \gamma \right) }{D_{1}\left( \gamma \right) }\left( \mathrm {\Delta }\gamma \right) ^{2} \right)= & {} .591+.163\times \left( 1+\frac{1}{2}\times -.234\times {(1)}^{2} \right) \\ {}= & {} .735. \end{aligned}$$

## Discussion

A frequently expressed concern is that different weighting schemes to penalize rater disagreements may result in substantially different coefficient values and conclusions about whether the categorized data are reproducible (De Raadt et al. 2021). The present study considered how a power parameter, commonly applied to define weighting schemes, affects a broad class of chance-corrected weighted agreement coefficients. We allowed for a continuum of infinitely many weighting schemes: Researchers may decide to follow popular choices (e.g., linear, quadratic, or identity weights) or use some other value of the real-valued power parameter that would better fit their data context. For example, they may decide that chosen categories that are one step apart should receive a specific weight and adjust the power parameter value to obtain the corresponding weighting scheme.

The “optimal” weighting scheme depends on the specific study context (Cohen 1968; Gwet 2014). Linear weights are a natural choice when there are no obvious arguments to deviate from penalization in proportion to the distance of rater disagreement. However, stricter weighting schemes (e.g., radical weights) may be better if even relatively small disagreements are serious, and more lenient weighting schemes (e.g., quadratic weights) may be better if only rather large disagreements are problematic. Although researchers may choose a specific weighting scheme for good reasons related to their data context, the choice is subjective and likely prone to abuse. For example, empirical studies most commonly use lenient quadratic weights (Vanbelle 2016). However, these studies usually provide little or no justification for this choice (Crewson 2005). Therefore, it is important to understand how the values of chance-corrected weighted agreement coefficients respond to changes in the power parameter. Furthermore, empirical studies should become more transparent.

The present study addressed these issues. First, we obtained theoretical results that help understand when and why chance-corrected weighted agreement coefficients are susceptible to the power parameter and in which direction. We provided necessary and sufficient conditions for the coefficient value to increase or decrease and the relationship to intensify or weaken as the power parameter increases. Furthermore, we decomposed these conditions into components that pairwise compare different category distances based on the ratio of observed to expected-by-chance frequency. For example, a larger ratio for the smaller distance than the larger distance contributes to a positive relationship between the power parameter and the coefficient value. We showed that the relationship is monotonic if the number of categories equals three or the ratio of observed to expected-by-chance frequency is monotonic in the category distance.

Second, we provided closed-form expressions for the first-order and second-order derivatives of chance-corrected weighted agreement coefficients with respect to the power parameter. We proposed the first-order derivative and the ratio of both derivatives as measures to quantify coefficient susceptibility and change in susceptibility as the power parameter changes. These summary measures give researchers a quick impression of the amount and type of dependence, such as positive or negative susceptibility and intensifying or weakening patterns. For example, suppose coefficient susceptibility turns out to be only moderate. In that case, the authors could use the measures to show that the obtained coefficient value does not strongly depend on the chosen weighting scheme. We found that positive but weakening coefficient susceptibility is most common. Thus, the coefficient value usually increases as the power parameter increases but tends to become more stable for higher values of the power parameter. For example, moving from identity to linear weights (i.e., from $\gamma =0$ to $\gamma =1)$ likely triggers a larger change in the coefficient value than an equal-sized step from linear to quadratic weights (i.e., from $\gamma =1$ to $\gamma =2)$.

Third, we showed how other researchers could use the coefficient value and derivatives for the reported value of the power parameter to obtain quite accurate estimates of the coefficient value for unreported values of the power parameter. These calculations are quick and easy (e.g., in Microsoft Excel or using a hand calculator), and they do not require access to the original data set. Especially this last property is valuable: Empirical studies often do not show their underlying data, particularly in settings with more than two raters, where the data no longer fit within a simple contingency table. Ideally, authors of empirical studies provide *both* arguments to justify their chosen weighting scheme *and* the derivative-based measures to allow others to recompute the coefficient value for flexible other choices of the power parameter.

The literature has proposed reference tables to interpret the values of chance-corrected (weighted) agreement coefficients in terms of high or low (Landis and Koch 1977). However, there is a broad consensus that more lenient weighting schemes require stricter thresholds, making such tables less useful (e.g., Warrens 2013). Although a lenient weighting scheme may not need stricter thresholds if it would fit the specific data context, correction is necessary if solid arguments for such a weighting scheme are lacking. Unfortunately, the literature offers little or no guidance on which stricter thresholds are appropriate to correct. Therefore, an alternative approach could be to apply the original thresholds to a recomputed coefficient value for a less lenient weighting scheme that the outside researcher considers more appropriate. Our proposed measures allow for recalculations that are usually accurate in the first two decimals. Furthermore, these measures help identify whether the issue of correcting is essential for the considered data set, which would be the case if the degree of coefficient susceptibility is high.

Although we considered a broad class of chance-corrected weighted agreement coefficients, future research could obtain the first-order and second-order derivatives and related conditions for coefficients with different structures. Examples include the weighted kappa for $R\ge 3$ raters and Gwet’s AC2. Furthermore, future research could extend the analysis to coefficient versions that allow for missing data, where raters may classify different subsets of items (Gwet 2014; Van Oest and Girard 2021). Another avenue for future research pertains to the drivers of coefficient susceptibility. For example, the present study found that agreement coefficients are often more susceptible to the power parameter in settings with more categories (i.e., higher *C*). However, other drivers may be present too. Relatedly, we considered 31 data sets from the literature. Future research could include more data sets to improve the representativeness of the sample and present meta-analytic generalizations.

## Supplementary Information

Below is the link to the electronic supplementary material.

Supplementary file 1 (ox 19 KB)

## References

[CR1] Agresti A (1988). A model for agreement between ratings on an ordinal scale. Biometrics.

[CR2] Banerjee M, Capozzoli M, McSweeney L, Sinha D (1999). Beyond kappa: A review of interrater agreement measures. Canadian Journal of Statistics.

[CR3] Bennett EM, Alpert R, Goldstein AC (1954). Communication through limited response questioning. The Public Opinion Quarterly.

[CR4] Brennan RL, Prediger DJ (1981). Coefficient Kappa: Some uses, misuses, and alternatives. Educational and Psychological Measurement.

[CR5] Brenner H, Kliebsch U (1996). Dependence of weighted kappa coefficients on the number of categories. Epidemiology.

[CR6] Cicchetti DV, Allison T (1971). A new procedure for assessing reliability of scoring EEG sleep recordings. American Journal of EEG Technology.

[CR7] Cohen J (1960). A coefficient of agreement for nominal scales. Educational and Psychological Measurement.

[CR8] Cohen J (1968). Weighted kappa: Nominal scale agreement with provision for scaled disagreement or partial credit. Psychological Bulletin.

[CR9] Conger AJ (1980). Integration and generalization of kappas for multiple raters. Psychological Bulletin.

[CR10] Cookson WOCM, De Klerk NH, Musk AW, Armstrong BK, Glancy JJ, Hobbs MST (1986). Prevalence of radiographic asbestosis in crocidolite miners and millers at Wittenoom, Western Australia. British Journal of Industrial Medicine.

[CR11] Crewson PE (2005). Reader agreement studies. American Journal of Roentgenology.

[CR12] De Raadt A, Warrens MJ, Bosker RJ, Kiers HAL (2021). A comparison of reliability coefficients for ordinal rating scales. Journal of Classification.

[CR13] Doornik J A (2007). Object-oriented matrix programming using Ox.

[CR14] Fleiss JL (1971). Measuring nominal scale agreement among many raters. Psychological Bulletin.

[CR15] Fleiss JL, Cohen J (1973). The equivalence of weighted kappa and the intraclass correlation coefficient as measures of reliability. Educational and Psychological Measurement.

[CR16] Fleiss J L, Levin B, Paik M C (2003). Statistical methods for rates and proportions.

[CR17] Graham P, Jackson R (1993). The analysis of ordinal agreement data: Beyond weighted kappa. Journal of Clinical Epidemiology.

[CR18] Guggenmoos-Holzmann I, Vonk R (1998). Kappa-like indices of observed agreement viewed from a latent class perspective. Statistics in Medicine.

[CR19] Gwet, K. L. (2014). *Handbook of inter-rater reliability: The definitive guide to measuring the extent of agreement among raters* (4th ed.). Advanced Analytics, LLC.

[CR20] Hand, D. J., Daly, F., Lunn, A. D., Conway, K. J., & Ostrowski, E. (1994). *A Handbook of Small Data Sets*. Chapman & Hall/CRC Press.

[CR21] Holmquist NS, McMahan CA, Williams OD (1967). Variability in classification of carcinoma in situ of the uterine cervix. Archives of Pathology.

[CR22] Janson H, Olsson U (2001). A measure of agreement for interval or nominal multivariate observations. Educational and Psychological Measurement.

[CR23] Kvålseth TO (2018). An alternative interpretation of the linearly weighted kappa coefficients for ordinal data. Psychometrika.

[CR24] Landis JR, Koch GG (1977). The measurement of observer agreement for categorical data. Biometrics.

[CR25] Maclure M, Willett WC (1987). Misinterpretation and misuse of the kappa statistic. American Journal of Epidemiology.

[CR26] Maria VAJ, Victorino RMM (1997). Development and validation of a clinical scale for the diagnosis of drug-induced hepatitis. Hepatology.

[CR27] Mielke PW, Berry KJ (2009). A note on Cohen’s weighted kappa coefficient of agreement with linear weights. Statistical Methodology.

[CR28] Mielke PW, Berry KJ, Johnston JE (2007). The exact variance of weighted kappa with multiple raters. Psychological Reports.

[CR29] Mielke PW, Berry KJ, Johnston JE (2009). Unweighted and weighted kappa as measures of agreement for multiple judges. International Journal of Management.

[CR30] Pratt JW (1964). Risk aversion in the small and in the large. Econometrica.

[CR31] Schuster C (2004). A note on the interpretation of weighted kappa and its relations to other rater agreement statistics for metric scales. Educational and Psychological Measurement.

[CR32] Scott WA (1955). Reliability of content analysis: The case of nominal scaling. Public Opinion Quarterly.

[CR33] Simonoff JS (2003). Analyzing Categorical Data.

[CR34] Sim J, Wright CC (2005). The kappa statistic in reliability studies: Use, interpretation, and sample size requirements. Physical Therapy.

[CR35] Soeken KL, Prescott PA (1986). Issues in the use of kappa to estimate reliability. Medical Care.

[CR36] Spitzer RL, Fleiss JL (1974). A re-analysis of the reliability of psychiatric diagnosis. British Journal of Psychiatry.

[CR37] Tinsley HEA, Weiss DJ (1975). Interrater reliability and agreement of subjective judgments. Journal of Counseling Psychology.

[CR38] Van Oest, R., & Girard, J. M. (2021). Weighting schemes and incomplete data: A generalized Bayesian framework for chance-corrected interrater agreement. *Psychological Methods*, online first publication. 10.1037/met0000412 34766799

[CR39] Van Oest R (2019). A new coefficient of interrater agreement: The challenge of highly unequal category proportions. Psychological Methods.

[CR40] Van Swieten JC, Koudstaal PJ, Visser MC, Schouten HJA, Van Gijn J (1988). Interobserver agreement for the assessment of handicap in stroke patients. Stroke.

[CR41] Vanbelle S (2016). A new interpretation of the weighted kappa coefficients. Psychometrika.

[CR42] Vanbelle S, Albert A (2009). A note on the linearly weighted kappa coefficient for ordinal scales. Statistical Methodology.

[CR43] Warrens, M. J. (2014). Power weighted versions of Bennett, Alpert, and Goldstein’s $$S$$. *Journal of Mathematics*. ID 231909, 9.

[CR44] Warrens MJ (2011). Cohen’s linearly weighted kappa is a weighted average of 2 $$\times $$ 2 kappas. Psychometrika.

[CR45] Warrens MJ (2012). Cohen’s quadratically weighted kappa is higher than linearly weighted kappa for tridiagonal agreement tables. Statistical Methodology.

[CR46] Warrens MJ (2012). Equivalences of weighted kappas for multiple raters. Statistical Methodology.

[CR47] Warrens MJ (2013). Conditional inequalities between Cohen’s kappa and weighted kappas. Statistical Methodology.

